# Cell-nonautonomous local and systemic responses to cell arrest enable long-bone catch-up growth in developing mice

**DOI:** 10.1371/journal.pbio.2005086

**Published:** 2018-06-26

**Authors:** Alberto Roselló-Díez, Linda Madisen, Sébastien Bastide, Hongkui Zeng, Alexandra L. Joyner

**Affiliations:** 1 Developmental Biology Program, Sloan Kettering Institute, New York, New York, United States of America; 2 Allen Institute for Brain Science, Seattle, Washington, United States of America; 3 Biochemistry, Cell and Molecular Biology Program, Weill Cornell Graduate School of Medical Sciences, New York, New York, United States of America; Lincolns Inn Fields Laboratory, United Kingdom of Great Britain and Northern Ireland

## Abstract

Catch-up growth after insults to growing organs is paramount to achieving robust body proportions. In fly larvae, injury to individual tissues is followed by local and systemic compensatory mechanisms that allow the damaged tissue to regain normal proportions with other tissues. In vertebrates, local catch-up growth has been described after transient reduction of bone growth, but the underlying cellular responses are controversial. We developed an approach to study catch-up growth in foetal mice in which mosaic expression of the cell cycle suppressor p21 is induced in the cartilage cells (chondrocytes) that drive long-bone elongation. By specifically targeting p21 expression to left hindlimb chondrocytes, the right limb serves as an internal control. Unexpectedly, left–right limb symmetry remained normal, revealing deployment of compensatory mechanisms. Above a certain threshold of insult, an orchestrated response was triggered involving local enhancement of bone growth and systemic growth reduction that ensured that body proportions were maintained. The local response entailed hyperproliferation of spared left limb chondrocytes that was associated with reduced chondrocyte density. The systemic effect involved impaired placental function and IGF signalling, revealing bone–placenta communication. Therefore, vertebrates, like invertebrates, can mount coordinated local and systemic responses to developmental insults that ensure that normal body proportions are maintained.

## Author summary

The coordination of organ growth is necessary to attain correct individual organ sizes and body proportions. While extensive studies in insects have revealed that both intra-organ and inter-organ communication mechanisms are involved in regulating organ growth, vertebrate studies have lagged behind. Here, we developed a new mouse model to examine cellular mechanisms underlying growth regulation after a developmental insult. The cell cycle suppressor p21 was expressed in the cartilage that drives growth of the long bones, targeting the left limb exclusively and leaving the right limb as an internal control. By triggering the insult during the last gestational week, we found that left–right limb symmetry was maintained due to the following 2 compensatory mechanisms: (1) hyperproliferation of the spared cells within the targeted cartilage, which indicates that these cells respond to a signal coming from the arrested cells, and (2) a growth reduction in the rest of the body, an effect that correlates with changes in the levels of placental insulin-like growth factor (IGF) signalling and that can be rescued by boosting placental efficiency. These results reveal that the response to developmental insults is quite evolutionarily conserved across species as well as open new avenues of future research for the development of therapies to treat growth disorders.

## Introduction

An important question in biology is how cells integrate intrinsic and extrinsic information such that their combined behaviours produce higher-order processes and structures, as seen during organogenesis and tissue repair. In *Drosophila* larvae, injured imaginal discs can undergo compensatory proliferation [1] as well as secrete an alarm signal that triggers both a systemic developmental delay and reduced growth of the spared imaginal discs [2–5]. Together, these processes allow the damaged tissue(s) to catch-up with other tissues, but the role of damaged versus undamaged cells remains controversial [6,7]. In vertebrates, systemic growth reduction after injury in a nonessential organ has not been reported. However, systemic catch-up growth has been described after transient impairment of whole-body growth [8–10], and local growth compensation can occur after unilateral manipulation of long bones within the limbs [11]. Tight control of inter-limb and limb–body proportions are critical for efficient locomotion and interaction with the environment, and therefore long bones are an excellent model for studies of growth regulation.

Growth of the long bones is driven by a process called endochondral ossification (reviewed in [12,13]). Once mesenchymal cells condense into the template of the skeletal elements, they differentiate into collagen II–expressing chondrocytes that go through sequential differentiation steps from bone ends to centre. Round resting chondrocytes give rise to flat proliferative cells that form columns and, after a few rounds of duplication, cease proliferation and start to differentiate into hypertrophic chondrocytes. These cells increase their volume as they progress towards the centre of the shaft, lay down a collagen X–rich extracellular matrix, and secrete factors that recruit vasculature and bone precursors (osteoblasts) from the perichondrium, a fibrous layer that wraps the cartilage [14,15]. Some hypertrophic chondrocytes die by apoptosis, while others transdifferentiate into osteoblasts [16,17]. Osteoblasts form the primary ossification centre by replacing the original matrix with bone. This process is later repeated at the ends of the bone (epiphyses), forming the secondary ossification centres. The so-called growth plate remains as a cartilage disc between the primary and secondary ossification centres and responds to both intrinsic and extrinsic factors that ultimately regulate bone length. For example, indian hedgehog (IHH), secreted by pre-hypertrophic chondrocytes, and parathyroid hormone-like peptide, secreted by resting chondrocytes, form a negative feedback loop that couples chondrocyte proliferation and differentiation (reviewed in [12,13]). This loop is the main conduit through which other local signals, such as fibroblast growth factors and bone morphogenetic proteins, exert their function, often impacting on the expression of key transcription factors. A number of systemic and local extrinsic signals (growth hormone, insulin-like growth factors [IGFs]) also play crucial roles in the modulation of chondrocyte activity and bone growth [18,19]. As per the regulation of growth after an insult, it has been proposed that bone catch-up growth is due to a cell-autonomous delay in the normal developmental decline of chondrocyte proliferation, such that when the insult is lifted, the formerly arrested chondrocytes retain a higher proliferative potential correlating with the stage at arrest [9,20]. It was suggested that a similar mechanism applies to other organs [21]. However, the possible contribution of unaffected cells has not been examined, which is important because a cell-autonomous mechanism does not account for cases in which catch-up growth is faster than expected for the observed maturation delay (reviewed in [13,22]).

Here, we developed new mouse models to transiently decrease long-bone growth in mice in order to determine the contributions of cell-autonomous and nonautonomous regulation during catch-up growth. Namely, we blocked proliferation in 50% of the cartilage chondrocytes that drive long-bone elongation, specifically in the left hindlimbs, such that the right limb remains as an internal control. Unexpectedly, left–right symmetry was maintained, revealing the deployment of compensatory mechanisms. Locally, we observed hyperproliferation of wild-type (WT) chondrocytes that mostly compensated for the lack of proliferation of their arrested neighbours. Systemically, a mild growth reduction affected the rest of the body, contributing to maintenance of limb–body proportions. In summary, our results reveal that long-bone catch-up growth shares some similarities with the response of imaginal discs to developmental insults in insects. We found that this response is mostly cell nonautonomous, representing a paradigm shift in the field that opens up new research avenues for basic and translational studies.

## Results

### An intersectional genetic approach enables inducible p21 misexpression preferentially in left limb chondrocytes

**Fig 1:**
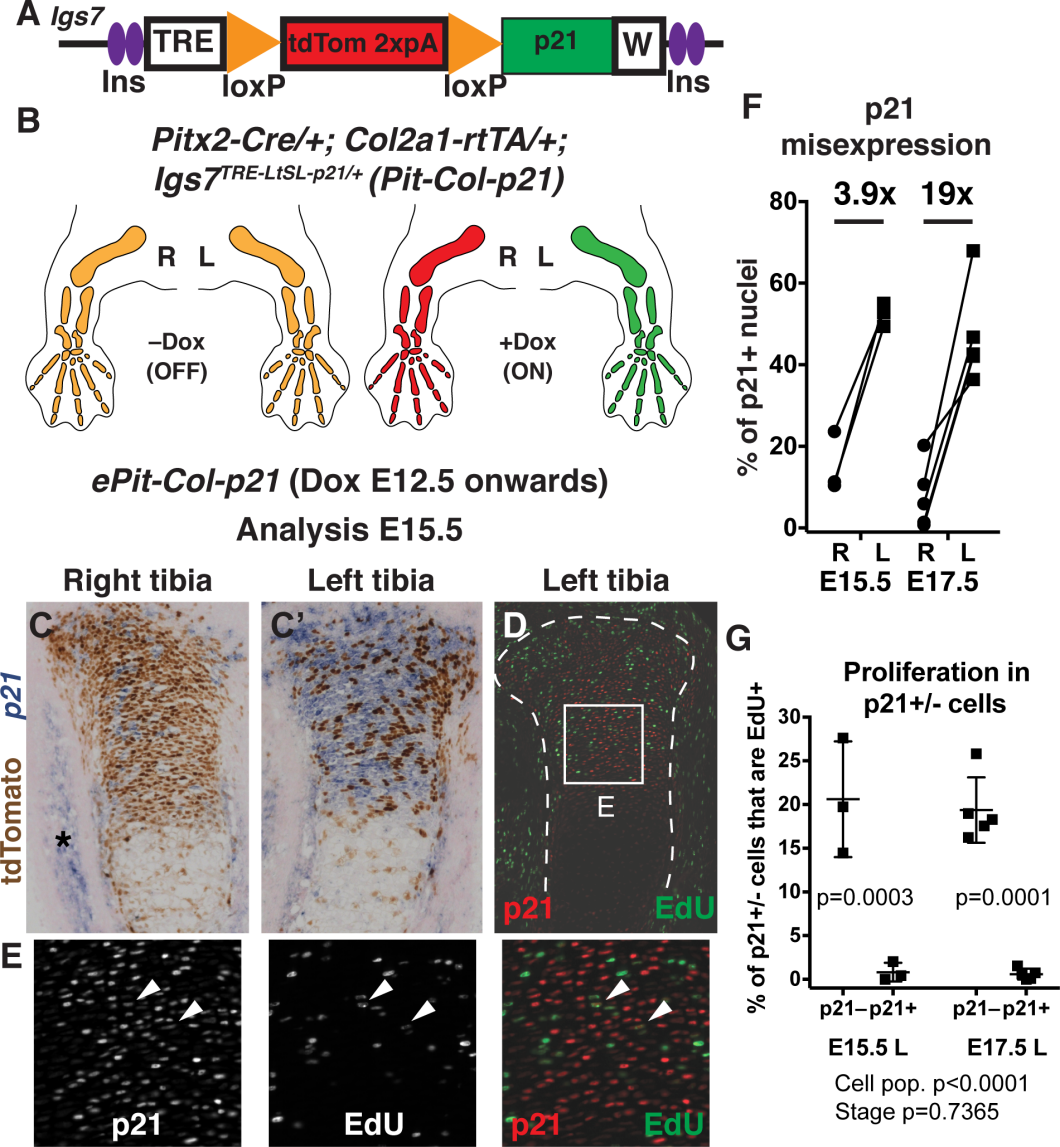
An intersectional genetic approach enables inducible p21 misexpression primarily in left limb chondrocytes. (A) *DRAGON-p21* allele in the *Igs7* locus. (B) Schematic showing *p21* expression driven by the left-specific *Pitx2-Cre* and cartilage-specific *Col2a1-rtTA* (*Pit-Col-p21*). (C–E) Expression of tdT protein and *p21* mRNA (panel C and C’) and p21 protein and EdU (panel D and E) at E15.5, with Dox administered at E12.5. *n =* 3. Box in panel D is magnified in panel E. Asterisk indicate endogenous *p21* expression. Arrowheads indicate rare double-positive cells. (F) Quantification of p21^+^ cells in the PZ of *ePit-Col-p21* proximal tibias, at E15.5 (*n =* 3) and E17.5 (*n =* 5). The average left/right fold-change is indicated. See also S3 Data. (G) Quantification of EdU incorporation in p21^+^ and p21^−^ cells of left *ePit-Col-p21* PZ of the cartilage, at E15.5 and E17.5 (*n =* 3 and *n* = 5). Comparison by 2-way ANOVA with Cell population and Stage as variables (*p*-values below graphs). *p*-Values for Sidak’s multiple comparisons post hoc test (between cell populations) are shown on the graph. For panel F and G, see S3 Data. 2xpA, transcriptional STOP; E, embryonic day; EdU, 5-ethynil-2’-deoxyuridine; Ins, insulator; PZ, proliferative zone; tdT, tdTomato; TRE, Tetracycline-responsive element; W, WPRE (mRNA-stabilizing sequence) followed by pA.

A major roadblock for studies of intra- and inter-organ growth regulation in mouse embryos has been a lack of models in which growth rate can be altered in a specific cell type within an organ, and ideally in only one of two paired organs, leaving the unmanipulated organ as an internal control. To address this deficiency, we devised new mouse models of inducible and transient growth inhibition in the left limb. We generated an *Igs7*^*TRE-LtSL-p21/+*^ allele, a variant of a double-conditional allele [23], to achieve doxycycline (Dox)-tuneable misexpression of the cell cycle suppressor *Cdkn1a* (*p21* hereafter) [24] in the cells in which activities of the bacteriophage recombinase Cre and of the (reverse) tetracycline transactivator ([r]tTA) intersect (Fig 1A and 1B). Due to a floxed tdTomato-STOP sequence (LtSL), expression of tdTomato (tdT) takes place in cells expressing (r)tTA but having no history of Cre activity, whereas *p21* is expressed in the cell population with a history of Cre and current (r)tTA activity (Fig 1A). We named the general type of allele Dox-controlled and Recombinase Activated Gene OverexpressioN (DRAGON). By combining the *DRAGON-p21* allele with an *asymmetric-Pitx2-enhancer-Cre* line expressing Cre in the precursors of the left limb mesenchyme (S1A–S1F Fig) [25] and a cartilage-specific *Col2a1-rtTA* line containing the reverse tetracycline transactivator under the control of a type II collagen promoter [26] (Fig 1B), Dox-dependent ectopic *p21* expression was achieved specifically in non-hypertrophic chondrocytes of the left limb cartilage elements (Fig 1C and 1C’). Consequently, any growth adjustment detected in the right limb of triple transgenic animals (*Pit-Col-p21*) when compared to control littermates must be due to activation of a systemic effect or inter-organ communication.

When Dox was administered from embryonic day (E) 12.5 until birth (*ePit-Col-p21* model), analysis at E14.5–E17.5 revealed the expected cartilage-exclusive expression of tdT, mainly in the right skeletal elements, and *p21* expression preferentially in the left limb cartilage, albeit in a mosaic fashion. For example, 36%–67% versus 0.8%–23% of chondrocytes were found to be p21^+^ in left versus right proximal tibia (S1G–S1K Fig, Fig 1C–1F; *n =* 3 E15.5; *n* = 5 E17.5 proximal tibias; *n* = 3 E17.5 proximal humerus). As we previously observed with the *Cre* transgene [19], the activity of Cre and therefore *p21* expression was more widespread in the left hindlimb than in the left forelimb (S1I–S1K Fig; only 22%–38% of chondrocytes were p21^+^ in the left proximal humerus). Therefore, we focused our initial analysis on the hindlimb. As expected, proliferation was inhibited in p21^+^ proximal tibia chondrocytes at E15.5 and E17.5 (Fig 1D and 1E and 1G; *n =* 3 and *n =* 5, respectively). Although a potential consequence of *p21* misexpression in proliferative zone (PZ) chondrocytes could have been their premature differentiation, we did not find precocious expression of chondrocyte maturation markers (e.g., *Ihh*, *Col10a1*, *Cdkn1c*, S2A and S2B Fig). Moreover, p21 expression did not induce cell senescence (monitored by expression of p19 and p16) by E17.5 (S2D Fig), nor did it hamper chondrocyte survival at E15.5 or E17.5 (S2E Fig). However, the normal expression domains of *Ihh*, *Cola10a1*, and *Cdkn1c* in (pre)hypertrophic chondrocytes (which do not express the transgene) appeared slightly fainter in the distal femur and proximal tibia cartilage, and RNA sequencing (RNA-seq) analysis of the combined proliferative and pre-hypertrophic zones revealed that their normalized counts were diminished in the left versus right cartilage (although only significantly for *Cdkn1c*. S2A and S2B Fig and S1 Data, S2 Data. See S6 Fig for description of the RNA-seq experiment). These results suggest that expression of p21 causes only a mild maturation impairment.

### Mosaic local proliferation blockade in the left limb cartilage slightly reduces bone growth but does not alter left–right limb symmetry

**Fig 2:**
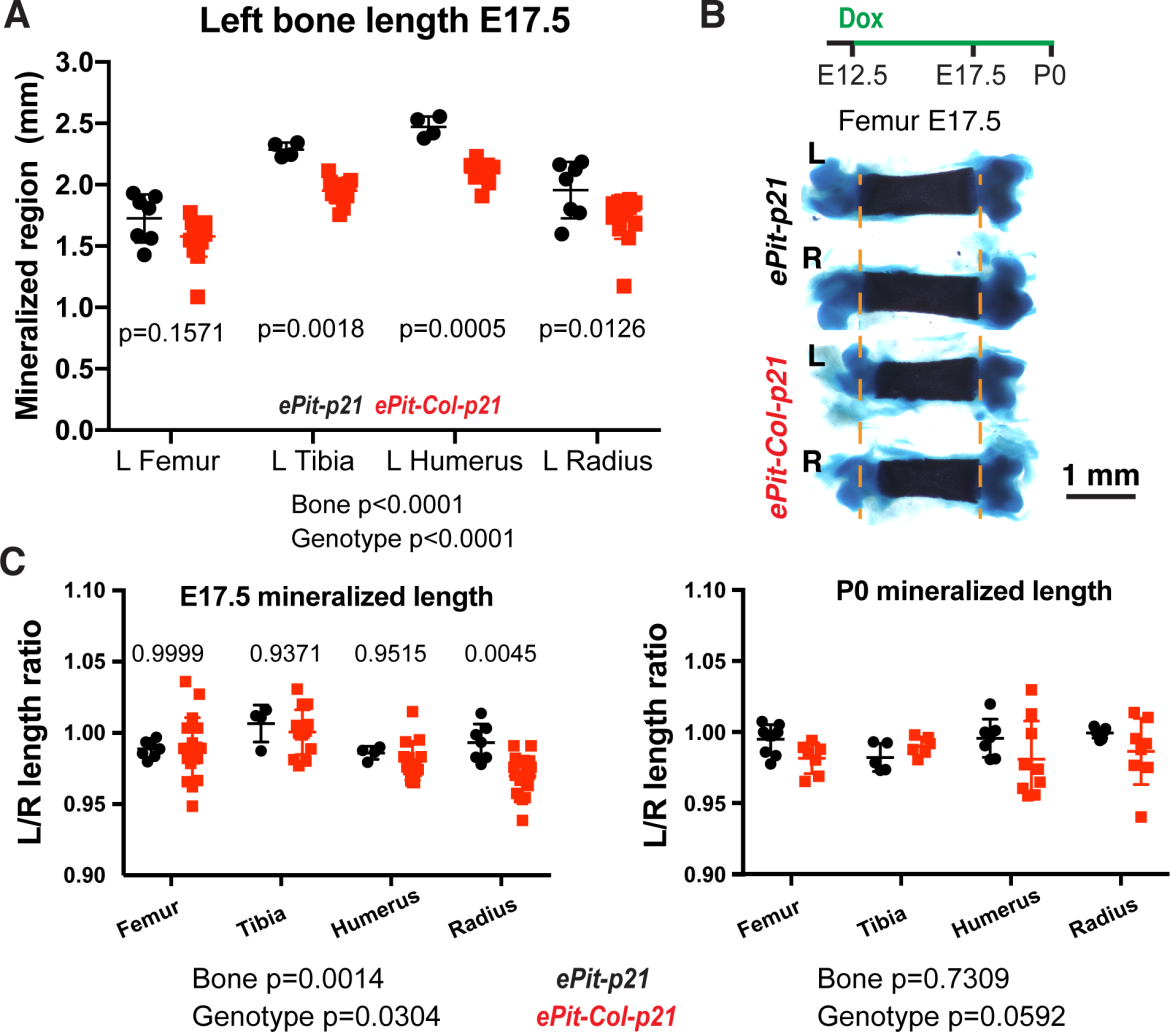
Mosaic proliferation blockade in the left limb cartilage slightly reduces bone growth but not left–right limb symmetry. (A) Absolute length of the indicated left bones of *ePit-p21* (*n =* 4–7 depending on the bone) and *ePit-Col-p21* (*n =* 11–15) E17.5 embryos. (B, C) Skeletal preparations (panel B) and quantification of the left/right ratio (panel C, mean ± SD) of the calcified region of the indicated bones at E17.5 (*n =* 4–7 *ePit-p21* and *n* = 11–15 *ePit-Col-p21* mice, depending on the bone) and P0 (*n =* 5–9, and 6–9). Dashed lines in panel B mark the ends of the mineralized region in control bones. In panel A and C, data were analysed by 2-way ANOVA with Genotype and Bone identity as variables. *p-*Values are shown below the graphs. For variables with significantly different measurements, Sidak’s post hoc test *p-*values are shown in the graph. For panel A and C, see S3 Data. E, embryonic day; P, postnatal day.

We next examined whether mosaic chondrocyte arrest altered left bone growth. As a first test, we compared the lengths of several left forelimb and hindlimb bones of *ePit-Col-p21* mice to *Pitx2-Cre; Igs7*^*TRE-LtSL-p21/+*^ control mice (*ePit-p21* hereafter). At E17.5, the left bones were 0.2–0.3 mm (approximately 10%) shorter in animals misexpressing p21 (Fig 2A, *n =* 7 for *ePit-p21*; *n* = 15 for *ePit-Col-p21* femora and radii; *n =* 4 and 11 for humeri and tibiae), indicating that blocking chondrocyte proliferation resulted in decreased bone growth. However, the effect was milder than expected, given that between one-third and two-thirds of chondrocytes were being arrested. This result suggested that compensatory mechanisms that minimized the impact of the p21 insult had been activated in the left limbs. Indeed, at E15.5 or E17.5, no major changes in the length of the proliferative or hypertrophic zones of the growth plate were found (S2F and S2G Fig).

We next took advantage of our unilateral approach that provides an experimental and a control limb within an animal and performed left–right intra-individual comparisons to determine the degree of asymmetry. Unexpectedly, most *ePit-Col-p21* bones measured at E17.5 or birth (P0) showed no obvious difference in their left/right length ratio compared to *ePit-p21* control littermates. The one exception was a transient small reduction in the size of the left radius compared to the right (Fig 2B and 2C, *n* ≥ 4 for *ePit-p21*; *n* ≥ 11 for *ePit-Col-p21* at E17.5; *n* ≥ 5 and 6 at P0). These results suggested that the right (i.e., control) bones in *ePit-Col-p21* mice responded to the impact of p21 expression in the left limbs via a systemic effect or inter-organ communication, such that their growth was reduced similarly to that of the left bones.

### Cell-nonautonomous compensation by spared neighbours in response to mosaic blockade of chondrocyte proliferation

**Fig 3:**
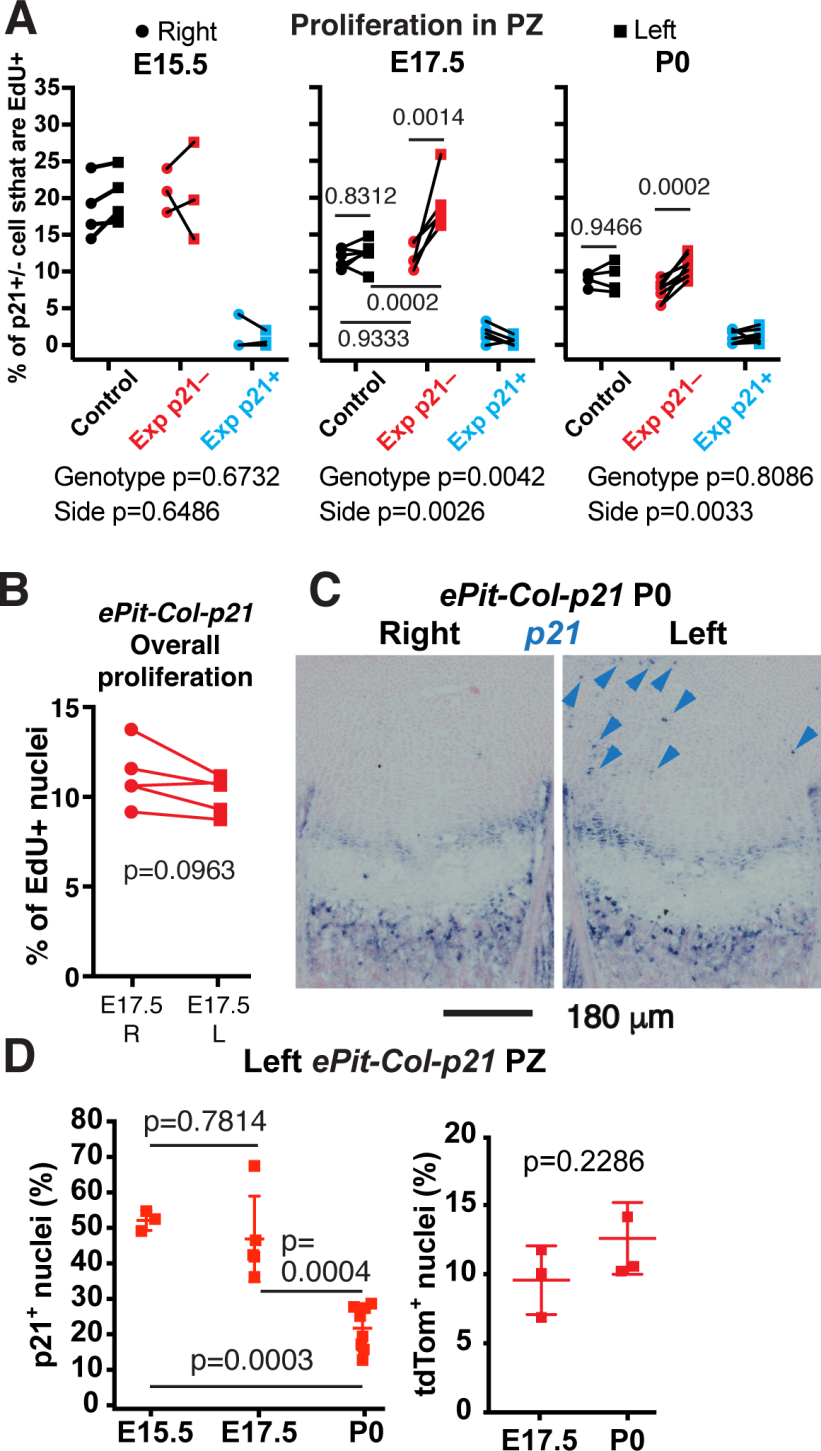
Cell-nonautonomous compensation by spared neighbours in response to mosaic blockade of chondrocyte proliferation. (A) % of p21^+^ or p21^−^ chondrocytes that have EdU^+^ nuclei in the PZ in the left and right proximal tibia of E15.5, E17.5, and P0 *ePit-p21* (Control, *n =* 4, 6, and 4) and *ePit-Col-p21* (Exp, *n =* 3, 5, and 8) embryos. p21^−^ cells from Control and Exp mice were compared by 2-way ANOVA with Side and Genotype as variables (*p-*values below graphs). For each significant variable, *p-*values for Sidak’s multiple comparisons post hoc test are shown in the graph. (B) % of EdU^+^ chondrocytes in the PZ of left and right proximal tibias of E17.5 *ePit-Col-p21* embryos, without distinguishing by p21 expression. Comparison by paired 2-tailed *t* test. (C–D) In situ hybridisation of p21 (panel C, arrowheads denote ectopic expression) and quantification of tdT and p21 (panel D) on sections of left *ePit-Col-p21* tibial PZs at E15.5, E17.5, and P0. *n =* 3, 5, and 8 for p21; 3 at each stage for tdT. The % of p21^+^ cells was compared by 1-way ANOVA (*p* < 0.0001). *p-*Values for Tukey’s multiple comparisons post hoc test are shown. The % of tdT^+^ cells (a proxy for rtTA activity) was compared by unpaired 2-tailed Mann-Whitney test. For panel A, B, and D, see S3 Data. E, embryonic day; EdU, 5-ethynyl-2’-deoxyuridine; Exp, Experimental; PZ, proliferative zone; rtTA, reverse tetracycline transactivator; tdT, tdTomato.

The compensatory response observed in the limbs of *ePit-Col-p21* mice could be due to both bone-intrinsic cellular mechanisms and the aforementioned apparent extrinsic regulation. We first tested for any organ-intrinsic responses and started by examining proliferation of the spared chondrocytes in the hindlimbs. Indeed, the left/right ratio of 5-ethynyl-2’-deoxyuridine (EdU) incorporation by p21^−^ chondrocytes was higher in experimental animals as compared with controls at E17.5 and P0 but not E15.5 (Fig 3A and S2H Fig), revealing cell-nonautonomous compensatory proliferation of p21^−^ cells in the presence of p21^+^ neighbours. Because p21^−^ cells did not differ in size from those of control mice (S2I Fig), the hyperproliferation of these cells at E17.5 likely contributes to the lack of a left-specific growth reduction in *ePit-Col-p21* embryos. In fact, overall EdU incorporation in left and right *ePit-Col-p21* PZs (without distinguishing between p21^+^ and p21^−^ cells), while tending to be slightly reduced, was not significantly different, indicating that the compensatory proliferation phenomenon is quite effective (Fig 3B). Moreover, the proliferative disadvantage of p21^+^ versus p21^−^ chondrocytes in the left limb of *ePit-Col-p21* mice resulted in dilution of p21^+^ chondrocytes from 45%–50% of PZ chondrocytes at E15.5 and E17.5 to approximately 20% at P0 (Fig 3C and 3D; *n =* 3, 5, and 8, respectively), and this depletion was not due to inactivation of rtTA activity (Fig 3D; *n =* 3 at E17.5; *n* = 3 at P0).

### Compensatory proliferation involves local cell interactions

**Fig 4:**
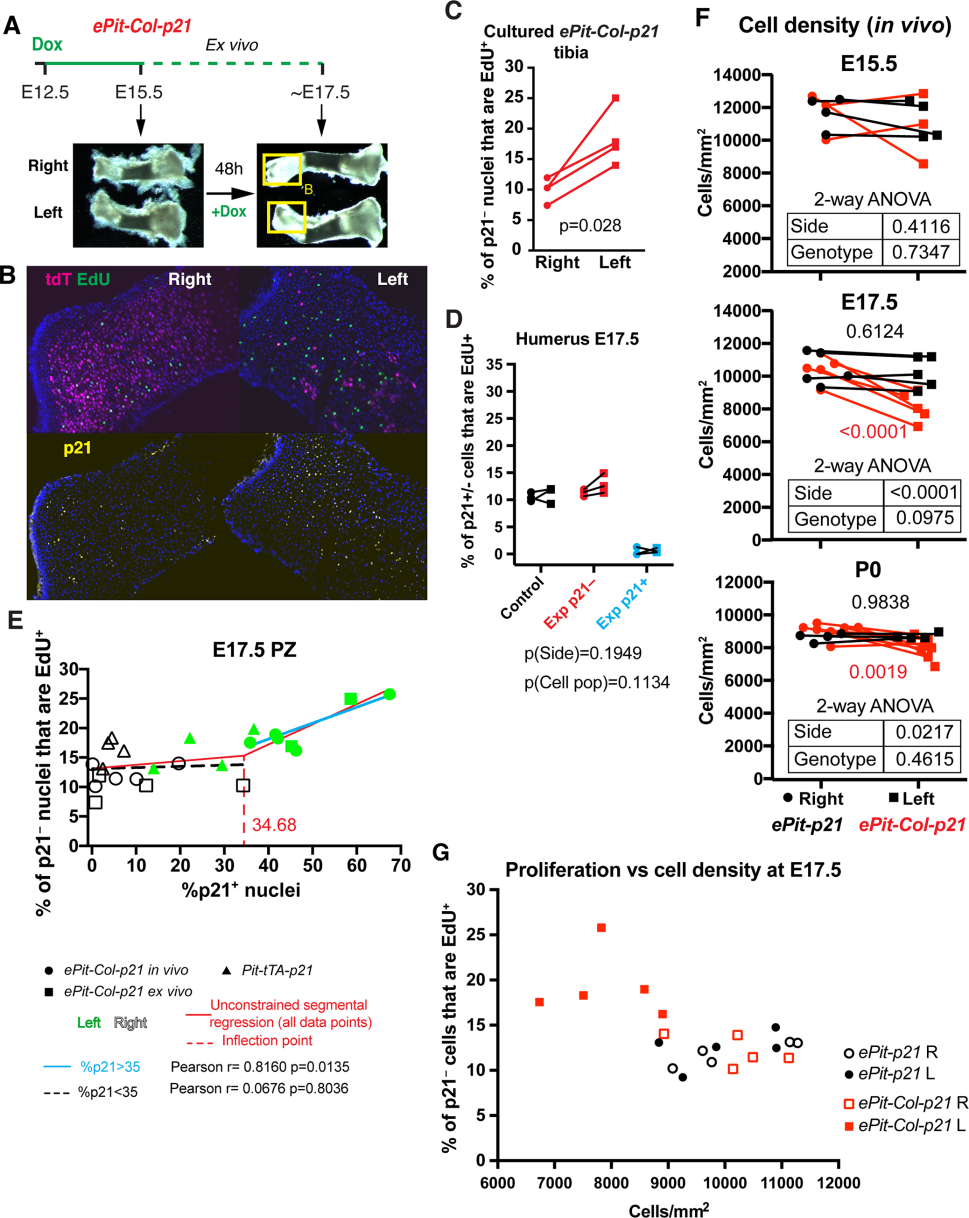
Compensatory proliferation is bone-intrinsic and takes place when chondrocyte density is lower than normal. (A) Summary of the ex vivo tibial culture experiment. The boxed regions correspond to the epiphyses shown in panel B. (B) Immunohistochemistry for the indicated molecules. (C) EdU quantification on distal PZ sections obtained from E15.5 *ePit-Col-p21* tibiae cultured for 2 d. *p-*Value for 2-tailed paired *t* test comparing left and right proliferative ratios of p21^−^ chondrocytes is shown (*n =* 4). The distal cartilage was quantified because the proximal one (bulkier) shows proliferation only in the periphery. (D) % of p21^+^ or p21^−^ chondrocytes that have EdU^+^ nuclei in the PZ in the left and right proximal humerus of E17.5 *ePit-p21* (Control, *n =* 3) and *ePit-Col-p21* (Exp, *n =* 3) embryos. p21^−^ cells from Control and Exp mice were compared by 2-way ANOVA with Side and Genotype as variables (*p-*values below graphs). (E) Correlation analysis between the extent of EdU incorporation in p21^−^ cells and the amount of p21^+^ nuclei in left and right PZ of *ePit-Col-p21* (*n =* 5 in vivo and *n* = 4 ex vivo) and *Pit-tTA-p21* tibial cartilage (*n =* 4) at E17.5. The inflection point revealed by unconstrained segmental regression was rounded up and used as a dividing threshold for the 2 correlation analyses (colour-coded). Pearson correlation coefficients and 2-tailed *p-*values are shown. (F) Comparison of chondrocyte density in the PZ of left and right *ePit-p21* and *ePit-Col-p21* proximal tibial cartilage at E15.5 (*n =* 4 and *n* = 3), E17.5 (*n =* 5 and *n* = 5) and P0 (*n =* 4 and 7) and analysed by 2-way ANOVA for Genotype and Side (*p-*values shown in the embedded tables). When *p* < 0.05 for these variables, colour-coded *p-*values for Sidak’s post hoc tests are shown. (G) EdU incorporation in p21^−^ chondrocytes of left and right PZ from E17.5 *ePit-p21* and *ePit-Col-p21* embryos (*n =* 5 each), plotted against cell density in the PZ. Note the sharp change in proliferation beyond 9,000 cells/mm^2^. For (C–G), see also S3 Data. Dox, doxycycline; E, embryonic day; EdU, 5-ethynyl-2’-deoxyuridine; Exp, Experimental; P, postnatal day; PZ, proliferative zone.

As a means to examine whether compensatory proliferation was only dependent on local cell–cell interactions, we cultured left and right E15.5 *ePit-Col-p21* tibiae (together in the same well) for 2 d with Dox, in the absence of surrounding mesenchyme (Fig 4A). We found that the distal tibia showed chondrocyte proliferation throughout all section levels, with very few or no senescent cells (S3A Fig, *n =* 3), whereas EdU incorporation was not detected in the inner core of the proximal tibia (bulkier than the distal one), probably because of insufficient nutrient diffusion. We therefore focused our analysis on the distal epiphysis. Similar to our findings in vivo, EdU incorporation in p21^−^ chondrocytes was significantly higher in the left as compared to the right cultured cartilages (Fig 4B and 4C), suggesting that compensatory proliferation is a bone-intrinsic phenomenon or at least does not require constant interaction with the surrounding mesenchymal tissues. As a control, we tested whether right cartilage proliferation was impaired due to the bone being cultured with the injured left tibia, since this would cause the impression of compensatory proliferation taking place in the left bone. We therefore cultured left and right tibiae from each embryo in different wells (S3B Fig) and found that EdU incorporation in distal right tibiae was not significantly different between bones cultured together (*n =* 4) or separately (*n =* 6) from the left bones.

### Compensatory proliferation requires a minimum threshold of p21^+^ chondrocytes

We next addressed whether the proportion of p21^+^ chondrocytes in the growing cartilage influences the extent of compensatory proliferation. Given that forelimb bones show a lower proportion of p21^+^ chondrocytes than hindlimb bones within each embryo analysed (S1K Fig; 55.4% ± 11.2% in tibia versus 32.8% ± 7.1% in humerus; *n =* 3; *p* = 0.0267 for paired *t* test), we first tested whether compensatory proliferation was triggered in the proximal humerus. We found that although there was a trend towards increased proliferation in left p21^−^ chondrocytes, the difference was not significant (Fig 4D; *n =* 3), suggesting that compensatory proliferation requires a minimum insult threshold to be triggered. Because intrinsic differences between forelimb and hindlimb bones might exist in regards to the compensatory proliferation response, we also tested the threshold hypothesis using only hindlimb bones. In order to induce p21 expression in fewer chondrocytes than in *ePit-Col-p21* mice, we made use of a newly generated *Col2a1-tTA* line (see Materials and methods) in place of the *Col2a1-rtTA* transgene, such that *p21* expression was achieved from approximately E12.5 onwards in the absence of Dox (*Pit-tTA-p21* model) (S4A Fig). tTA expression in this line is less extensive than rtTA in the *Col2a1-rtTA* line, as *p21* misexpression was detected in fewer left tibial chondrocytes than in *ePit-Col-p21* left tibia (30%–40% at E15.5, 15%–35% at E17.5, 10%–20% at P0; S4A Fig). Consistent with our prediction of a threshold being needed, compensatory proliferation was not detected in the *Pit-tTA-p21* model (S4B and S4C Fig). To further investigate whether a minimum insult threshold is required to trigger increased proliferation, we calculated the correlation coefficient between the percentage of p21^+^ chondrocytes and the extent of proliferation in the PZ from left and right *ePit-Col-p21* (in vivo and ex vivo) and *Pit-tTA-p21* tibiae, at E17.5 (or E15.5 plus 2 d ex vivo). Segmental linear regression analysis revealed that the extent of EdU incorporation by p21^−^ chondrocytes did not correlate with the proportion of p21^+^ neighbours when this proportion was below 35%, but beyond this threshold, there was a linear correlation between both parameters (Fig 4E; *n =* 26 bones). These results suggest that compensatory proliferation is due to a signal produced in proportion to the number of arrested chondrocytes, that the signal needs to reach a certain threshold to be effective, and that it remains active until at least P0 despite the dilution of p21^+^ chondrocytes.

### Compensatory proliferation is possibly related to epiphyseal cell density

We next asked whether additional cellular changes occur in the left limbs of *ePit-Col-p21* mice that could contribute to the growth compensation and potentially correlate with the number of insulted chondrocytes. Because an alteration in cell density can influence organ size, we tested whether cell density was changed in the PZ of *ePit-Col-p21* mice. That was indeed the case, and we found a temporal association between the occurrence of compensatory proliferation in the *ePit-Col-p21* model (i.e., at E17.5 and P0 but not E15.5) and statistically significant reduction of cell density in the left PZ as compared to the right (Fig 4F). Notably, left and right PZ cell densities were not significantly different at any stage in *ePit-p21* mice (Fig 4F, *n =* 12). Moreover, in line with the threshold hypothesis, we found that, at E17.5, there was a certain value of cell density below which EdU incorporation sharply increased in p21^−^ chondrocytes (Fig 4G, *n =* 20 bones).

### Mosaic local proliferation blockade in chondrocytes of the left limb results in a systemic growth reduction

**Fig 5:**
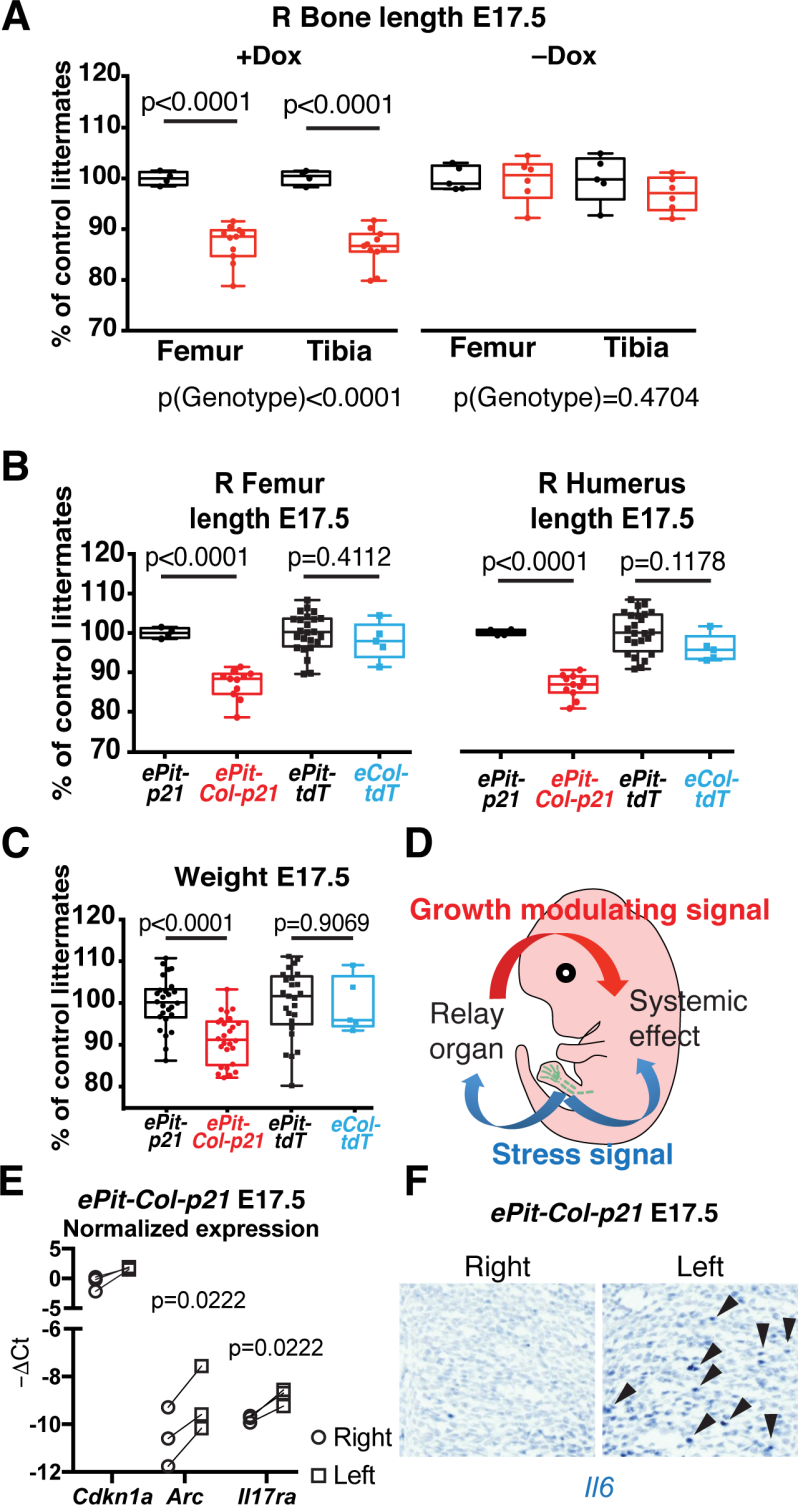
Mosaic local proliferation blockade in chondrocytes of the left limb results in systemic growth reduction. (A) Right femur and tibia length (normalized to the average *ePit-p21* littermate) from E17.5 embryos treated with Dox (*n =* 4 *ePit-p21* and *n* = 11 *ePit-Col-p21*) or untreated (*n =* 5 and *n* = 6). Comparison by 2-way ANOVA with Genotype and Bone identity as variables. *p-*Values for Genotypes are shown below graphs; *p-*values for Sidak’s post hoc test shown on graph. (B–C) Box and whisker plots for normalized bone length (panel B) and weight (panel C) of *ePit-Col-p21* and *Col2a1-rtTA; Igs7*^*TRE-tdT/+*^ embryos (*eCol-tdT*, expressing tdT in all cartilage elements), compared to their controls (*ePit-p21* and *ePit-tdT*) by unpaired *t* tests. *p-*Values corrected for multiple comparisons (Holm-Sidak method) are shown. For panel B, *n =* 22 *ePit-p21*, *n* = 26 *ePit-Col-p21*, *n* = 25 *ePit-tdT*, and *n* = 5 *eCol-tdT*). For panel C, *n =* 4, 11, 24, and 5. (D) Model of the systemic growth response after local chondrocyte arrest triggers a stress signal. (E–F) qRT-PCR (panel E) and in situ hybridisation (panel F) for the indicated transcripts in the proliferative plus pre-hypertrophic zone from *ePit-Col-p21* embryos. Panel E shows one of 2 independent experiments with 3 distinct biological replicates each (total *n* = 6). The –ΔCt (relative to *Gapdh*) for each stress-related transcript was compared by a paired *t* test (left versus right). In panel F, *n =* 2 E15.5, *n* = 4 E16.5, and *n* = 6 E17.5 embryos (arrowheads denote *Il6* expression). For panel A–C and E, see also S3 Data. Dox, doxycycline; E, embryonic day; qRT-PCR, quantitative real-time polymerase chain reaction; tdT, tdTomato.

Given our initial finding that left and right limb bones exhibit reduced growth in *ePit-Col-p21* embryos as compared to *ePit-p21* littermates (Fig 2), we next investigated whether there was a systemic response to the p21 insult in the left limbs. Because the growth phenotype is quite mild (an approximately 10% reduction), we first confirmed the finding by measuring micro-computerized tomography (μCT)-generated 3D reconstructions instead of flat micrographs (S5A and S5B Fig; *n =* 7 for *ePit-p21* and *n* = 13 for *ePit-Col-p21* embryos). We found a very good correlation between both types of measurements (S5C Fig, *n =* 80 bones) and therefore used flat micrographs for all measurements in the study. We first tested whether the growth reduction affected the whole body. We found that, in addition to a decrease in right bone length, body weight of E17.5 and P0 *ePit-Col-p21* mice—but not E15.5 or E16.5 embryos—was approximately 10% lower than in *ePit-p21* littermates (Fig 5A–5C, S5D Fig). Furthermore, the bone-length and weight effects required Dox treatment and therefore *p21* expression (Fig 5A–5C). As control experiments, we confirmed that there was no leakiness of the intersectional misexpression strategy (S5E Fig) that could account for the systemic growth reduction and that misexpression of tdT in all chondrocytes did not cause a systemic growth reduction (Fig 5B and 5C). Our results thus revealed a whole-body response to an insult in a specific tissue in mice, similar to what has been described in *Drosophila* larvae [2–5].

**Fig 6:**
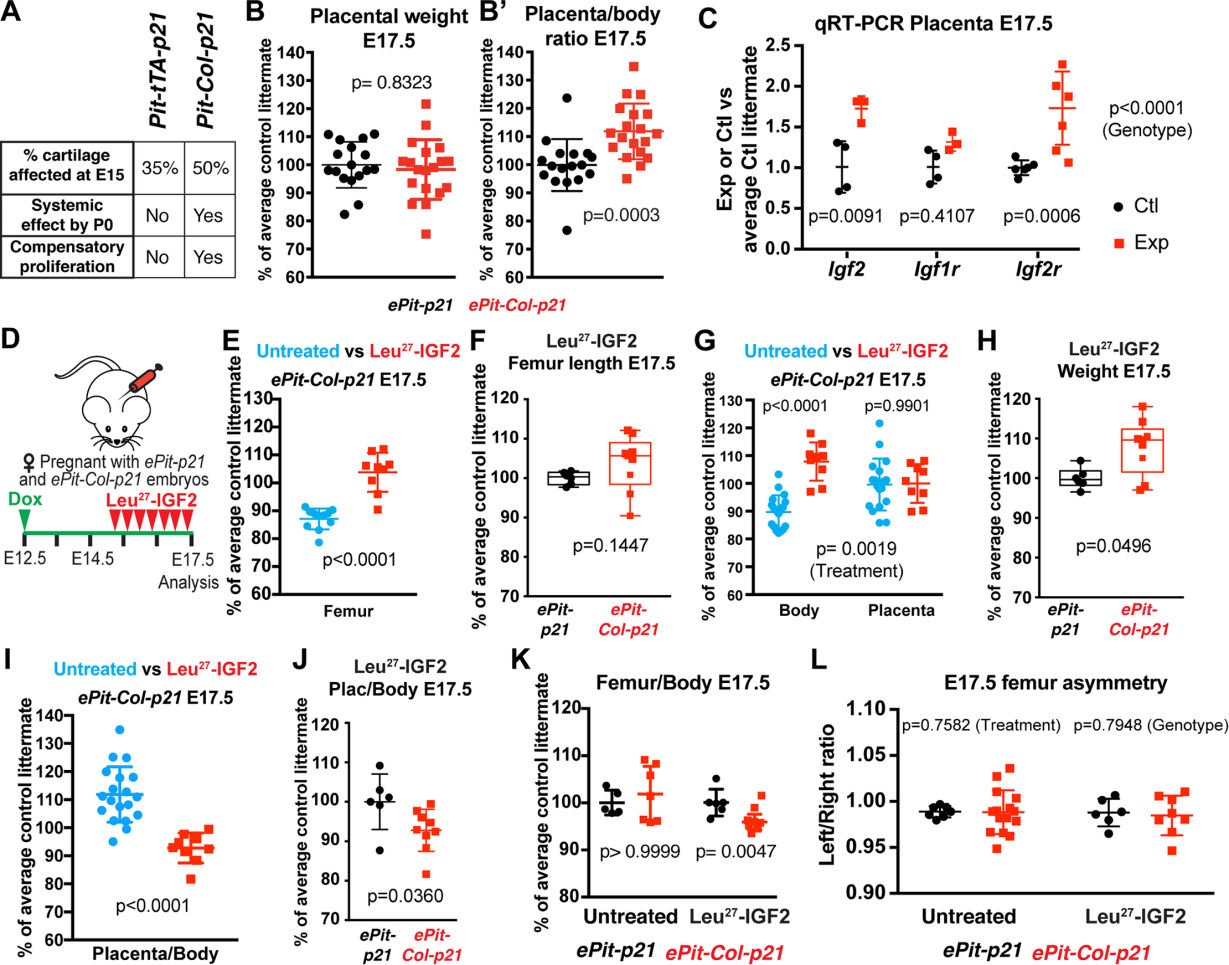
The systemic effect involves impaired placental function and its rescue leads to altered limb–body proportions. (A) Summary of the characteristics and outcomes of the different injury models. (B, B’) Placental weight (panel B) and placenta/body weight ratio (panel B’) of *ePit-Col-p21* embryos (*n =* 19), normalized to the average of *ePit-p21* littermates (*n =* 17) at E17.5 and compared by 2-tailed unpaired Mann-Whitney test. (C) qRT-PCR for *Igf2*, *Igf1r*, and *Igf2r* (with *Tbp* as reference gene) in the placenta of E17.5 *ePit-Col-p21* and *ePit-p21* embryos, normalized to the average value of control littermates (*n =* 4 Control and *n* = 3 Exp, except *Igf2r*, for which 6 of each were analysed). Two-way ANOVA with Gene and Genotype as variables was used. *p-*Values for Genotype (right) and for Sidak’s post hoc tests (on graph) are shown. (D) Pregnant females were injected 3 times each day with Leu^27^-IGF2 in an attempt to improve placental efficiency. (E–F) Characterization of femur length at E17.5 in Leu^27^-IGF2-treated versus untreated *ePit-Col-p21*-embryos (panel E) and between control and experimental embryos within treated litters (panel F); *n =* 11 untreated; *n* = 9 treated *ePit-Col-p21* embryos; *n* = 6 treated *ePit-p21* ones. Unpaired 2-tailed Mann-Whitney test was used. (G–H) Same as panel E and F, for body and placental weight, *n =* 6 treated *ePit-p21* embryos; *n* = 19 untreated and 9 treated *ePit-Col-p21* embryos. Two-way ANOVA with Conceptus part and Treatment as variables was used in panel G. *p-*Values for Treatment (bottom) and for Sidak’s post hoc tests (top) are shown. Unpaired 2-tailed Mann-Whitney test was used in panel H. (I–J) Same as panel E and F, for placenta/body weight ratio, normalized to the average control littermate (*n =* 6 treated *ePit-p21* embryos; *n* = 19 untreated and 9 treated *ePit-Col-p21* embryos). Unpaired 2-tailed Mann-Whitney test was used. (K) Femur length/body weight ratio of untreated or Leu^27^-IGF2-treated E17.5 *ePit-p21* and *ePit-Col-p21* embryos, normalized to the average control littermate (*n =* 5 untreated and *n* = 6 treated Control; *n* = 6 untreated and *n* = 8 treated Exp embryos). For each treatment, comparisons by unpaired Mann-Whitney test are shown. (L) Left/right ratio of femur length for E17.5 *ePit-p21* and *ePit-Col-p21* embryos from Leu^27^-IGF2-treated (*n =* 6 Control and *n* = 8 Exp) and -untreated litters (*n =* 7 Control and *n* = 15 Exp). *p-*Values (2-way ANOVA) for Treatment and Genotype are shown. For panel B through L, see also S3 Data. E, embryonic day; Exp, Experimental; IGF2, insulin-like growth factor 2; qRT-PCR, quantitative real-time polymerase chain reaction.

In order to characterize the cartilage response, we performed an RNA-seq experiment to identify differentially expressed genes (DEGs) between left and right cartilage (PZ plus pre-hypertrophic region of proximal and distal tibia and femur) of single *ePit-Col-p21* embryos at E17.5 (S6A–S6E Fig, S1 Data and S2 Data). Indeed, overrepresentation analysis of the DEG (adjusted *p*-value ≤ 0.05) showed enrichment of several pathways related to stress and immune responses in the left cartilage (S6F Fig). In particular, we found several stress-related transcripts that shared a similar left–right pattern of expression within each embryo (S6G Fig) and verified their enrichment in the left cartilage by quantitative real-time polymerase chain reaction (qRT-PCR) (Fig 5E) or in situ hybridisation (Fig 5F). *Relaxin1*, the closest homologue to *dilp8*, the recently identified [3,27] alarm gene in fly, was not expressed at significant levels in either limb (S6E Fig), suggesting that the mechanism that links the local insult with a systemic response has diverged during evolution. With regards to the relationship between the extent of insult and the induction of a systemic response, *Pit-tTA-p21* mice did not trigger a systemic growth defect at E17.5 or P0 (S4D and S4E Fig, summary in Fig 6A), suggesting that the systemic growth reduction, like compensatory chondrocyte proliferation, is only triggered when a certain insult threshold is surpassed in the targeted cartilage.

### The systemic growth reduction of *ePit-Col-p21* embryos involves impaired placental function and when it is rescued, limb–body proportions are altered

We reasoned that the most likely foetal organ to control systemic growth by responding to a circulating alarm signal is the placenta because in rodents it produces higher IGF levels than any other organ [28] and is considered the main organ controlling foetal growth [29], whereas hepatic IGFs regulate systemic growth mainly after weaning [18]. Placental weight was not diminished in *ePit-Col-p21* embryos (*n =* 19) as compared to *ePit-p21* controls (*n =* 17), such that the placenta/body weight ratio was increased (Fig 6B). This result suggests that placental efficiency is reduced in response to the left-cartilage p21 insult. To determine the status of placental IGF signalling, we tested the expression of several pathway members by qRT-PCR and found that *Igf2* levels were increased in the placentas of *ePit-Col-p21* embryos as compared to *ePit-p21* controls, whereas the levels of *Igf1r* did not vary significantly (Fig 6C; *n =* 3 experimental and *n* = 4 control). Increased expression of *IGF2* by the placenta has been seen as part of a placental stress response triggered by prenatal insults such as alcohol exposure, which is also associated with placental functional impairment, increased placental/body weight ratio, and foetal growth restriction [30,31]. Therefore, one possible interpretation of our results is that the left limb cartilage stress response is relayed to the placenta, which then indirectly impacts on foetal growth. Perhaps explaining why increased IGF2 expression does not correlate with enhanced embryo growth, we found an increase in the level of *Igf2r* (Fig 6C; *n =* 6 experimental and *n* = 6 control), which encodes a decoy receptor that can decrease local IGF2 availability [32]. Furthermore, inhibition of IGF2R has been shown to boost placental efficiency [33].

As a means to test whether the systemic growth reduction in *ePit-Col-p21* embryos was due to impaired IGF signalling within the placenta, we injected pregnant dams (from E15.25 to E17.25, 3 times per day, Fig 6D) with an IGF2 analogue (Leu^27^-IGF2) that does not cross the placental barrier, can only bind IGF2R (and not IGF1R), and thus was shown to increase placental efficiency [33]. Body and placental weight and femur length of *ePit-Col-p21* embryos were compared between litters that were either treated or not treated with Leu^27^-IGF2 to determine the degree to which body/organ size was rescued, and they were also compared with *ePit-p21* embryos within treated litters to determine whether Leu^27^-IGF2 differentially affected experimental and control embryo growth. Boosting placental function led to the following results:

1. Femur length of treated *ePit-Col-p21* embryos was significantly increased compared to untreated experimental embryos (*n =* 9 and *n* = 11, respectively), and within treated litters, femur length was not significantly different between *ePit-Col-p21* and *ePit-p21* littermates (*n =* 6), demonstrating preferential rescue of the mutant embryos (Fig 6E and 6F).
2. Whereas placental weight remained unchanged following Leu^27^-IGF2 administration, body weight of treated *ePit-Col-p21* embryos was significantly increased compared to untreated experimental embryos (*n =* 9 and *n* = 19), and within-litter comparison revealed that body weight was not only rescued but also slightly increased beyond that of *ePit-p21* littermates (*n =* 6), suggesting that *ePit-Col-p21* embryos are especially sensitized to the systemic growth factors produced by the placenta (Fig 6G and 6H). Consequently, the placenta/body ratio was notably reduced in treated versus untreated *ePit-Col-p21* embryos, to levels even lower than in treated *ePit-p21* littermates (Fig 6I and 6J).
3. Because the body size was “over-rescued” as compared to the bones, the femur/body weight ratio of rescued *ePit-Col-p21* embryos was diminished compared to *ePit-p21* littermates or untreated litters (Fig 6K, *n =* 5 untreated and *n* = 6 treated *ePit-p21* embryos; *n* = 6 untreated and *n* = 8 treated *ePit-Col-p21*). Taken together, these results suggest that, in the presence of local growth defects, a systemic growth reduction is necessary to maintain limb–body proportions. Unexpectedly, rescue of the systemic effect did not result in left–right asymmetry in *ePit-Col-p21* embryos (Fig 6L), indicating that a specific decrease in growth of the unmanipulated limb, which is independent of placental function, contributes to the maintenance of left–right symmetry upon a unilateral insult.

## Discussion

### A holistic view of compensatory responses triggered by developmental insults

In summary, our results show that when the embryonic long bones experience mosaic inhibition of chondrocyte proliferation, an adaptive growth response can be triggered that involves cell-nonautonomous local mechanisms and systemic changes during the time frame of the insult, such that body proportions are maintained. We refer to this new type of catch-up growth that happens during an on-going insult as ‘adaptive growth’ (S7 Fig). Our finding that a local compensatory response occurs during the insult and involves cell-nonautonomous mechanisms is distinct from previous models that proposed that compensation occurs after the insult is lifted and is cell-autonomous [9,11,20]. Therefore, our results introduce a new conceptual framework for interpreting studies of perturbed long-bone growth. Furthermore, the experimental approach we devised for the study of growth regulation in mice makes a strong case for using unilateral perturbation approaches when studying bilateral organs. Although a local response such as compensatory proliferation or reduced cell density could have been unveiled with a mosaic bilateral injury, a subtle body-weight effect would likely be ascribed to the reduced size of all limbs and not to inter-organ communication. Indeed, the hint that prompted us to explore inter-organ communication was the observed reduction in the unmanipulated limb between experimental and control mice. Below, we discuss the potential mechanisms and evolutionary conservation of local and systemic responses to developmental injury.

### Compensatory proliferation

We have shown that a few days into mosaic inhibition of proliferation affecting >35% of chondrocytes of the left limb, spared chondrocytes undergo increased proliferation, such that the overall proliferative rate in the left cartilage almost matches that of the right limb. We propose the following order of events, based on correlative data from our study:

1. In response to a reduction in the number of chondrocytes produced, extracellular matrix production is increased (and thus cell density is reduced). As a consequence, the amount of cartilage scaffold being laid down is not reduced, and thus endochondral ossification can proceed at an almost normal rate. This proposed response fits with stereological studies that showed that, in growth plates with low proliferative rates, it is mainly the production of extracellular matrix that contributes to bone growth [34]. In this regard, the small decrease in chondrocyte density seen in *Pit-tTA-p21* mice could explain why these mice do not show increased left–right asymmetry despite lacking compensatory proliferation and systemic growth reduction (S4F and S4G Fig).
2. When the insult is extensive enough for cell density to drop below a certain threshold, compensatory proliferation is triggered in spared chondrocytes, to an extent proportional to the number of affected chondrocytes. Because we found that the extent of compensatory proliferation does not linearly correlate with cell density—but it does with the proportion of p21^+^ chondrocytes—we posit that density plays a permissive rather than an instructive role and that stress signals emanating from p21^+^ chondrocytes are needed as well. These stress signals could be diffusible molecules or cell–cell interactions, and they could act either in short range, affecting only cells in their proximity, or in long range, via a self-propagated travelling wave [35] and leading to a whole-organ (i.e., community) effect (S7A and S7B Fig). Moreover, although our ex vivo experiments suggest that compensatory proliferation is a bone-intrinsic process, we cannot rule out that tissues such as the cartilage–bone interface or the perichondrium (also present in the cultures) play a role in modulating compensatory proliferation. To distinguish between all these possibilities, new insult models that allow triggering of focal insult domains of variable size and positions will be required.
3. Once p21^+^ chondrocytes have been replaced by p21^−^ cells and the threshold cell density has recovered, compensatory proliferation stops, such that overgrowth is not generated.

### Systemic growth reduction

Our results reveal a mild but consistent systemic growth reduction (approximately 10%) in response to a local insult in the cartilage. We propose that the stress response generated in the left cartilage is somehow communicated to the placenta, which in turn systemically reduces growth (S7 Fig). We suggest the following 2 mechanisms that could account for the injured cartilage–placenta communication:

1. Stress signals are produced locally—either by p21^+^ chondrocytes, p21^−^ chondrocytes, the perichondrium, or cells at the cartilage–bone interface—are released into circulation, and, when they surpass a threshold, impact on placental efficiency (S7C Fig). It was recently shown that a subpopulation of natural killer (NK) cells that are transiently abundant in the decidual region of the placenta can promote placental function and foetal growth [36]. If the function of these cells was impaired by the circulating stress signals in *ePit-Col-p21* embryos, this could negatively impact on placental function and explain the systemic growth reduction.
2. There is a size-monitoring system that detects impaired long-bone growth and leads to a systemic growth reduction that allows the impaired organ to keep up with the rest of the body. Such a mechanism would likely require a central integrator where size-for-age information is stored and compared to actual organ size, but as of yet, there is almost no evidence for such a system (see discussion in [13]).

With regards to potential growth correction treatments, it would be important to determine whether all *ePit-Col-p21* organs are equally reduced or whether the musculoskeletal system (which is especially dependent on IGF signalling) is primarily affected. Resolution of the latter question is currently difficult because the embryos are too small for individual organs to be weighed reliably. Volumetric analyses using mesoscopic techniques such as optical projection tomography [37] on embryos expressing fluorescent reporters in the tissue of interest will be necessary to achieve the necessary level of resolution.

### Left–right limb crosstalk

An unexpected result of our study is that when placental function is boosted in *ePit-Col-p21* concepti through maternal Leu^27^-IGF2 treatment, long-bone growth is not enhanced to the same extent as body weight, resulting in a reduction in the ratio of bone length to body weight (Fig 6). Given that the right cartilage templates are not experiencing the same p21 insult as the left ones, the dampened response of the right skeletal elements to the systemic rescue suggests that the insult in the left cartilage influences growth of the right limb through some sort of left–right crosstalk. A similar crosstalk has been previously proposed in studies of amphibian limb regeneration, in which it was shown that amputation of the contralateral limb at the same rostrocaudal level as the originally amputated limb reduced the regenerative rate of the latter, whereas ipsilateral or contralateral amputation at a different rostrocaudal level did not [38]. Moreover, a study of tibial fracture repair in young rats showed that the healing environment of a fractured bone triggers the release of growth-promoting signals in the growth plate of the fractured bone and that the same signalling is induced in the contralateral growth plate [39]. As previously proposed [38], the most obvious candidate system to mediate crosstalk between the left and right limbs is the nervous system. While the exact mechanism remains to be determined, a recent study showed that peripheral sympathetic nerves might inhibit bone growth in response to sustained social stress [40]. Regardless of the mechanism, these results suggest that the observed systemic growth reduction in *ePit-Col-p21* embryos is a combination of 2 effects: reduced growth efficiency of the contralateral bones in response to the left-specific insult, and impairment of placental function that affects the rest of the body.

### Evolutionary conservation of compensatory mechanisms

Collectively, our results reveal that the processes leading to coordination of growth within and between organs to achieve normal proportions upon developmental insults are conserved across metazoans. However, the magnitude of the contributions of local and systemic mechanisms likely varies across phyla because the extent of the systemic growth reduction observed in mice seems to be less extreme than in *Drosophila*, with the caveat that different insults or tissues could elicit distinct responses. The exact underlying mechanisms also vary because we did not observe up-regulation of the *dilp8* homologue *Relaxin1* in the insulted cartilage. Different molecular mechanisms aside, the compensatory response in vertebrates shares some features with the response in insects, such as our finding that the injured tissue is able to catch up despite being exposed to an environment that stunts growth of the rest of the body. One explanation for this result is that local compensatory proliferation overrides a systemic effect. We further speculate that if the same ‘alarm’ signal were to trigger both the intrinsic and systemic mechanisms following injury, this would provide an evolutionarily advantageous strategy to achieve robust coordination of organ growth.

While many unknowns remain in the field of organ growth and repair, further exploration of the mechanisms revealed by this study will open exciting new avenues for basic and translational research and lead to an understanding of human growth disorders.

## Materials and methods

### Ethics statement

All animal studies were performed under an approved Institutional Animal Care and Use Committee mouse protocol (#07-01-001) according to MSKCC institutional guidelines.

### Study design

To correct for interlitter variability when studying the effect of p21 misexpression on systemic growth, we normalized each measurement from an experimental animal (*ePit-Col-p21* or *Pit-tTA-p21*) to the average measurement for its control littermates (*ePit-p21* or *Pit-p21*). This is important because the absolute measurements vary significantly between litters, in part because they differ in exact developmental stage, number of embryos, and age of the mother [36]. For paired measurements, the use of left/right ratios allowed for intra-individual normalization. For each experiment, the minimum sample size was estimated using an online tool (http://powerandsamplesize.com/Calculators), based on the average SD observed in pilot experiments, to achieve an effect size of 0.03 in the left/right bone length ratio, 0.5 in the left/right ratio of EdU incorporation, or 10% in normalized systemic measurements, with a power of 0.8 and a 95% CI. In Fig 5B and 5C, 2 embryos (one from the *ePit-Col-p21* and one from the *eCol-tdT* populations) were abnormally small, possibly dead, and were excluded from the analysis. For comparison of qualitative expression, a minimum of 2 specimens per stage and 5 across several stages were used. The investigator measuring bone length was blinded to the treatment/genotype of the specimens. No blinding was done for other measurements. No randomization was used for animal processing.

### Statistics

When data were available for control and experimental, a normalized measurement (left/right ratio or percentage of average control mice) was calculated for both. Between different time points, the normalized measurements were compared by multiple unpaired *t* test with Holm-Sidak correction for multiple comparisons. Within the same time point, comparisons were done by an unpaired Mann-Whitney test (1 variable and 2 conditions), by 1-way ANOVA (1 variable and ≥3 conditions), or by 2-way ANOVA (2 variables and 2 or more conditions) following a matched (paired) design when possible (indicated when not). When left and right measurements were compared within experimental animals only, paired 2-tailed *t* test was used. For all ANOVA, alpha = 0.05. All relevant parameters for the statistical tests can be found in S1 Table. When parametric tests were used, data normality was confirmed by Shapiro-Wilk test and equality of variance by F-test. Prism7 software (Graphpad) was used for most analyses. Most graphs show individual values and mean ± SD, unless otherwise indicated.

### Animals

To generate the *Igs7*^*TRE-LtSL-p21*^ mouse line, the *NruI*-STOP-loxP-tdTomato-*SnaBI* fragment in the *Ai62(TITL-tdT) Flp-in* replacement vector [23] was replaced by a custom *NruI*-tdTomato-STOP-loxP-*MluI-HpaI-SnaBI* cassette, to generate an empty DRAGON vector. A PCR-amplified Kozak-Cdkn1a cassette was subsequently cloned into the *MluI* and *SnaBI* sites to generate the *DRAGON-p21* vector. This vector was then used for recombinase-mediated cassette exchange into *Igs7-*targeted G4 ES cells [23]. Two successfully targeted clones were injected into C2J blastocysts to generate chimeras, obtaining 27 chimeric males (out of 30 born) with 75% to 100% chimerism. Two males from each clone were crossed to BL6 albino mice (Charles River, Wilmington, MA) to assess germline transmission and to establish the new mouse lines. To generate the *Col2a1-tTA* line, a *Kozak-tTA* fragment was PCR-amplified from plasmid *pEnt L1L3 tTA-3* (Addgene plasmid #27105, gift of Edward Hsiao) and cloned into a vector containing the regulatory region of mouse *Col2a1* obtained from plasmid *p3000i3020Col2a1* [41]. Backbone-free vector DNA was injected into FVB zygotes to generate transgenic lines. Four out of 11 founders transmitted the *Col2a1-tTA* allele. The progeny of one of those (founder number 92) expressed the tTA faithfully in the highest percentage of chondrocytes and was bred with *Pitx2-Cre* animals to generate breeders for the experiments.

*Col2a1-tTA* mice were maintained on an outbred Swiss Webster background and genotyped using primers *Col2a1-F* (CCAGGGTTTCCTTGATGATG) and *tTA-R* (GCTACTTGATGCTCCTGATCCTCC) and a standard PCR program with 55°C annealing temperature. The *Pitx2-Cre* [25] (kind gift of Dr. H. Hamada), *Col2a1-rtTA* [26] (kind gift of Dr. K. Posey), *Ai9* (*R26*^*CAGGS-LSL-tdTomato*^) [42], and *Ai62* (*Igs7*^*TRE-LSL-tdTomato*^) [23] mouse lines were maintained on an outbred Swiss Webster background and genotyped as previously described. *Igs7*^*TRE-LtSL-p21*^ animals were genotyped like *Ai62* mice. *Pitx2-Cre/Cre; Col2a1-(r)tTA/+* females and males homozygous for the conditional misexpression allele were crossed to generate experimental and control animals. Noon of the day of vaginal plug detection was considered E0.5. The equivalent of E19.5 is referred to as P0.

### Dox treatment

Dox hyclate (Sigma) was added to the drinking water at a final concentration of 1 mg/ml, with 1% sucrose to increase palatability.

### Tibial culture

A previously described protocol [43] was slightly adapted to culture embryonic long bones. Briefly, E15.5 tibiae were obtained from the embryos of Dox-treated pregnant females, dissected free of as much soft tissues as possible in ice-cold PBS, and then cultured (at 37°C, 5% CO_2_) in 24-well plates with serum-free DMEM (Gibco) containing 0.2% Bovine Serum Albumin (BSA), 0.5 mM L-glutamine, 40 U/ml penicillin/streptomycin (Gibco), 0.05 mg/ml ascorbic acid (Sigma), and 1 mM betaglycerophosphate (Sigma). The medium additionally contained 1 ng/μl Dox to maintain transgene expression. After 2 d, the bones were incubated with 10 μM EdU for 90 min, then fixed in PFA and processed for histological analysis.

Note that after 2 d, we consistently observed growth of 19% to 23% in control limbs as compared to the original length. This is less than in vivo (approximately 87% growth between E15.5 and E17.5), and the main difference seemed to be at the level of the proximal cartilage, which does not proliferate, likely due to insufficient diffusion of nutrients because it is larger than the distal cartilage. We therefore focused our analysis on the distal cartilage, which at these stages is expected to contribute one-third of total growth [44], i.e., approximately 29%, quite similar to the observed growth.

### Leu^27^-IGF2 injections

Human Leu^27^-IGF2 (GroPep, Australia) was prepared at 500 ng/μl in sterile 0.01 N HCl solution and kept at 4°C in between injections. From E15.25 to E17.25, the pregnant dam was subcutaneously injected every 8 h, for a total dose of 1 μg/g of body weight per day.

### Skeletal preparations and measurements

Staining of cartilage and bone was performed as described [45]. Bone length was measured on digital micrographs using the Line tool in Adobe Photoshop. Unless otherwise indicated, only the mineralized region was measured.

### Micro-CT scans and measurements

Whole femora and tibiae were scanned using a Scanco μCT 35 (Scanco Medical, Brüttisellen, Switzerland) system. Six-μm voxel size, 45 KVp, 0.36-degree rotation step (180-degree angular range), and a 400-ms exposure per view were used for the scans, which were performed in air. Scanco μCT software (HP, DECwindows Motif 1.6) was used for 3D reconstruction and viewing of images. After 3D reconstruction, ‘Distance 3D’ tool was used for measuring the length of the ossified region. Three measurements were taken and the average derived for each bone. The observer was blinded to the genotype of the mouse.

### Sample processing for histology

Mouse embryos were euthanized by hypothermia in cold PBS. Mouse pups were euthanized by decapitation after hypothermia-induced analgesia. Knees (or isolated full tibiae and femora) were dissected out, skinned, and fixed by immersion in 4% paraformaldehyde (PFA; Electron Microscopy Sciences) in PBS for 2 d at 4°C. After several washes with PBS, the tissue was then cryoprotected, first by brief incubation with a solution of 15% sucrose and then 30% sucrose in PBS for at least 4 h at 4°C, and then embedded in Cryomatrix (Thermo) using dry-ice-cold isopentane (Sigma). The knees were oriented sagittally and facing each other, with the tibiae on the bottom of the block (i.e., closest to the blade when sectioning). Serial 7-micron sections were collected with a Leica Cryostat on Superfrost slides, allowed to dry for at least 30 min, and stored at −80°C until used. For all histological techniques, frozen slides were allowed to reach room temperature in a closed box, and Cryomatrix was washed away for 15 min with warm PBS (37°C).

### Immunohistochemistry and TUNEL

Sections were incubated in citrate buffer (10 mM citric acid, 0.05% Tween 20 [pH 6.0]) for 15 min at 90°C, allowed to cool down, washed with PBSTx (PBS containing 0.1% Triton X-100), blocked with 5% BSA in PBSTx for 30 min at room temperature, and incubated with the primary antibody over night at 4°C (see list of antibodies below). After PBSTx washes, incubation with Alexa647- and/or Alexa555-conjugated secondary antibodies (Molecular Probes; 1/500 in PBSTx with DAPI) was performed for 1 h at room temperature. After PBSTx washes, the slides were mounted with Fluoro-Gel (Electron Microscopy Sciences). For TUNEL staining, endogenous biotin was blocked after antigen retrieval using the Avidin/Biotin blocking kit (Vector #SP-2001), and TdT enzyme and Biotin-16-dUTP (Sigma #3333566001 and #11093070910) were subsequently used following manufacturer instructions. Biotin-tagged DNA nicks were revealed with Alexa488- or Alexa647-conjugated streptavidin (Molecular Probes, 1/1000) during the incubation with the secondary antibody.

Antibodies (host species, vendor, catalogue number, dilution) included tdT (rabbit polyclonal, Rockland #600-401-379, 1/500), p21 (rabbit polyclonal, Santa Cruz Biotechnology #sc-471, 1/300), p19^Arf^ (rat monoclonal, clone 12-A1-1, Novus Biologicals #NB200-169, 1/100), and p16-INK4A (rabbit polyclonal, Proteintech #10883-1-AP, 1/300).

### In situ hybridisation

The protocol described in [46] was followed. For embryos and newborns, samples were not decalcified. Except for *Col2a1*, *Col10a1*, and *Ihh* (provided by Dr. Licia Selleri), the templates for most riboprobes were generated by PCR from embryonic cDNA, using primers containing the SP6 or T7 RNA polymerase promoters. Sequence of the primers is available upon request. After purification of the PCR product (Qiagen PCR purification kit), DIG-labelled probes were transcribed following manufacturer instructions (Roche), treated with DNAase for 30 min, and purified by LiCl-mediated precipitation in alcoholic solvent. Probes were kept at −80°C in 50% formamide (Fluka). For immunohistochemistry after in situ hybridisation, sections were incubated in citrate buffer (10 mM citric acid, 0.05% Tween 20 [pH 6.0]) for 15 min at 90°C, allowed to cool down, washed with PBSTx, and incubated with 1% H_2_O_2_ in PBSTx for 1 h to block endogenous peroxidases. After BSA blocking and primary antibody incubation, endogenous biotin was blocked using Avidin/Biotin Blocking kit (Vector #SP-2001), and then the slides were incubated with a biotinylated secondary antibody. A brown precipitate was obtained using a peroxidase-coupled streptavidin-biotin complex (Vectastain Elite ABC Kit, Vector #PK-6100) and DAB substrate (Vector #SK-4100), following manufacturer instructions.

### Imaging

For in vivo experiments, sagittal sections of the knees were imaged, focusing on the region between both menisci and analysing at least 2, and typically 4, sections per limb. For cultured distal tibiae, frontal sections were used because they allow for better identification of the different epiphyseal regions. The transition between round (resting) and flat (columnar) nuclei, roughly describing an arch between the upper point of the wedges formed by the groove of Ranvier, was chosen as the start of the PZ, while the transition towards bigger, more spaced nuclei (pre-hypertrophic) was chosen as the end of the PZ. The point where pericellular matrix is sharply reduced around enlarging chondrocytes was considered as the start of the HZ, while the end of the HZ was marked as the distal end of the last intact chondrocyte. Bright-field and fluorescence images were taken on a Zeiss inverted microscope (Observer.Z1) using Axiovision software (Zeiss). Mosaic pictures were automatically reconstructed from individual 10× (bright-field) or 20× (fluorescence) tiles.

### EdU incorporation

Five mg/ml EdU in PBS was injected (50μg/g body weight, s.c for pups, i.p. for adults and pregnant females) 1.5 h before euthanizing the mice. EdU was detected using the Click-iT Alexa488 Imaging Kit (Invitrogen, C-10337) once the immunohistochemistry and/or TUNEL staining were finished on the same slides. The fraction of nuclei that were positive for EdU, p21, or tdT in the PZ of the cartilage was determined using ImageJ or CellProfiler, followed by manual curation.

### Cell density analysis

The PZ was cropped from 20×-imaged sections of left and right experimental and control proximal tibial cartilage, stained for DAPI, p21, and EdU. The area of the region of interest (PZ) was measured in pixels using the Histogram tool in Adobe Photoshop and converted into mm^2^ using the resolution and scale information. The DAPI channel was segmented and quantified using Cell Profiler. Cell density was calculated as the number of chondrocytes per area unit.

### Cell size analysis

The PZ was cropped from 20×-imaged sections of left and right *ePit-Col-p21* proximal tibial cartilage. tdT^+^ chondrocytes were segmented and counted, and their individual area was measured using Cell Profiler.

### RNA isolation and analysis

The distal left or right femoral and proximal tibial cartilage from E17.5 *Pit-Col-p21* embryos was dissected in cold PBS, the condyles and hypertrophic zones removed using a microknife, and the perichondrium removed by a combination of collagenase type II treatment (Worthington, 2 mg/ml in DMEM, 2 min at room temperature) and mechanical dissection. Left and right cartilage fragments from each embryo (number 1, 2, and 3) were kept in separated tubes and flash-frozen in liquid nitrogen. RNA was extracted using Trizol (Invitrogen) and a mechanical tissue disruptor.

### RNA-seq

High-quality RNA was deep sequenced (≥50 million paired-end reads) by the New York Genome Center. Aligned reads were analysed using DESeq2 tool in R. A paired design was used, such that left and right comparison was performed for each specimen, which minimized the effect of sequencing batch and interspecimen variability. DEGs were obtained using a threshold of adjusted *p*-value ≤ 0.05. NMF library tools were used to generate heatmaps. Enrichment analysis was performed using DAVID [47] and WebGestalt [48].

### qRT-PCR

cDNA was synthesized from purified RNA using iScript reverse transcriptase (RT) as described by the manufacturer (Bio-Rad). Each target was amplified in triplicate to obtain an average per sample, using SYBR Green (Applied Biosystems) on a StepOnePlus real-time PCR system (Applied Biosystems). Primer sequences are shown in Table 1. Negative controls (no template) and no-RT cDNA controls were included for each primer/sample combination. Relative expression on each sample was calculated by the 2^−ΔCT^ method, with *Gapdh* (for cartilage) or *Tbp* (for placenta) as a reference.

**Table 1:** Sequence of the oligonucleotides used for qRT-PCR.

Primer name	Sequence 5’ → 3’
qPCR Cdkn1a F	CCTGGTGATGTCCGACCTG
qPCR Cdkn1a R	CCATGAGCGCATCGCAATC
qPCR Arc F	AAGTGCCGAGCTGAGATGC
qPCR Arc R	CGACCTGTGCAACCCTTTC
qPCR Il17ra F	AGTGTTTCCTCTACCCAGCAC
qPCR Il17ra R	GAAAACCGCCACCGCTTAC
qPCR Gapdh F	CCAATGTGTCCGTCGTGGATCT
qPCR Gapdh R	GTTGAAGTCGCAGGAGACAACC
qPCR Igf2 F	GTGCTGCATCGCTGCTTAC
qPCR Igf2 R	ACGTCCCTCTCGGACTTGG
qPCR Igf1r F	GTGGGGGCTCGTGTTTCTC
qPCR Igf1r R	GATCACCGTGCAGTTTTCCA
qPCR Igf2r F	TGAATGGTGATCCTTGCCCTC
qPCR Igf2r R	CCGGTAGCTGTTGGTCTGTC
qPCR Tbp F	GGGAGAATCATGGACCAGAA
qPCR Tbp R	GATGGGAATTCCAGGAGTCA

Abbreviation: qRT-PCR, quantitative real-time polymerase chain reaction.

## Supporting information

S1 Data Data for S2 Fig and S6 Fig. **RNA-seq data from left and right *ePit-Col-p21* growth plates.** Left (L) and right (R) proliferative and pre-hypertrophic zones from 3 different E17.5 *ePit-Col-p21* embryos were dissected and sequenced. Growth plates from femur and tibia were pooled. Normalized counts are shown. The original numbering (#386–388) was changed to 1–3.
(TXT)

S2 Data Data for S6 Fig. **List of DEGs between left and right *ePit-Col-p21* cartilage at E17.5.** The DESeq2 tool (adjusted *p* ≤ 0.05) was used to obtain the list.
(TXT)

S3 Data Data for Figs 1–6 and S1–S6 Figs. Individual values of the measurements presented throughout the study.
(XLSX)

S1 Table Parameters of the statistical tests used in this study. (XLSX)

S1 Fig Characterization of the left limb-specific intersectional approach to induce transient growth defects. (A–F’) *Pitx2-Cre* females were crossed with Ai9 males to characterize the specificity of Cre-mediated labelling. Seven-μm sections from left and right hindlimbs are shown at 2 different stages: E12.5 (A–D) and E18.5 (E–F’), *n =* 4 for each stage. Boxed regions in panel E and panel F are shown in E’, (E”, and F’. Most of the red signal on right limbs corresponds to autofluorescent blood cells. (G–H’) Dynamics of tdT and CDKN1A (p21) activation in *ePit-Col-p21* embryos, 1 d (G, G’, *n =* 2) and 2 d (H, H’, *n =* 3) after Dox administration to the pregnant female. Boxed regions in panel G and H are shown in G’ and H’. Note that activation of the transgene starts to be detectable 1 d post Dox administration, but it is not complete until 2 d post Dox. Asterisks indicate autofluorescent cells. Of note, the *Pitx2-Cre* allele is consistently left-predominant only when inherited from the female. (I–J”) Same as above, but E17.5 elbow sections are shown. (K) Intra-individual comparison of the proportion of p21^+^ nuclei in the left proximal humerus versus left proximal tibia PZ (*n =* 3). See also S3 Data. *p-*Value for 2-tailed paired *t* test is shown. Cre, recombinase from P1 bacteriophage; Dox, doxycycline; E, embryonic day; PZ, proliferative zone; tdT, tdTomato.
(TIF)

S2 Fig Histological, molecular, and cellular characterization of the effects of p21 misexpression. (A–C) The expression of chondrocyte maturation markers *Cdkn1c*, *Col10a1*, and *Ihh* is not ectopically triggered by p21 misexpression (panel A, B), but their expression is qualitatively and quantitatively diminished in the left cartilage (panel C, normalized counts and adjusted *p-*value from the RNA-seq experiment of S6 Fig are shown). For panel C, see S1 Data as well. (D–E) Misexpression of p21 does not lead to cell senescence in the experimental cartilage at E17.5 (panel D, monitored by p16 and p19 expression, *n =* 3), nor to ectopic cell death at E15.5 or E17.5 (panel E, arrows indicate TUNEL+ cells, *n =* 5). (F) Hematoxylin–eosin staining of E15.5 femora and E17.5 proximal tibiae from *ePit-Col-p21* embryos. (G) Comparison of the length of the left and right proliferative and hypertrophic zones (PZ and HZ) of the femora from *ePit-Col-p21* (*n =* 4) and *ePit-p21* embryos (*n =* 3) at E15.5 (2-way ANOVA with Genotype and Side as variables was used, and *p-*values are shown). (H) Left/right ratios of EdU^+^ incorporation in the PZ of *ePit-p21* and *ePit-Col-p21* embryos at E15.5 (*n =* 4 and *n* = 3), E17.5 (*n =* 5 and *n* = 5), and P0 (*n =* 4 and *n* = 8). Comparison by 2-way ANOVA for Genotype and Stage (*p-*values below graphs). *p-*Values for Sidak’s post hoc test are shown in the graphs. (I) Cell size of tdT^+^ (i.e., p21^−^) chondrocytes was characterized for *ePit-Col-p21* embryos at E17.5 (*n =* 10, see Materials and methods). Representative pictures of left and right PZ are shown. No significant difference between left and right distribution was found (*p-*value for 2-tailed unpaired Mann-Whitney test for ranks is shown). For panel G–I, see S3 Data. E, embryonic day; HZ, hypertrophic zone; PZ, proliferative zone; RNA-seq, RNA sequencing.
(TIF)

S3 Fig Characterization of the tibial culture system. (A) After 2 d of culture, the distal tibial cartilage does not show signs of senescence, as shown by lack of p16 immunostaining (*n =* 3). (B) Right tibiae show the same extent of proliferation regardless of whether they are cultured together (*n =* 4) or separated (*n =* 6) from the contralateral tibia. See also S3 Data.
(TIF)

S4 Fig Compensatory proliferation and systemic growth reduction are not detected by birth when *p21* is expressed in less than 35% of chondrocytes. (A) Left: schematic of the new *Col2a1-tTA* allele. See ref. [41] for details on the *Col2a1* regulatory region used. In the absence of Dox, the tTA is activated around E12.5 (detected by a germline-recombined reporter *Ai62* allele) [23]. Right: percentage of p21^+^ chondrocytes in the PZ of left proximal tibia of *Pit-tTA-p21* embryos unexposed to Dox, at E15.5, E17.5, and P0 (*n =* 3, 4, and 3). Comparison by 1-way ANOVA (*p* = 0.0368), followed by Tukey’s post hoc tests (shown). (B) Left/Right ratio of EdU incorporation in PZ chondrocytes of *Pit-tTA* and *Pit-tTA-p21* mice at E15.5 (*n =* 3 each), E17.5 (*n =* 4 each), and P0 (*n =* 3 each). Comparison by 2-way ANOVA for Genotype and Stage (*p-*values below graphs). (C) Percentage of p21^+^ or p21^−^ chondrocytes that have EdU^+^ nuclei in the PZ in the left and right tibias of E17.5 *ePit-p21* (Control) and *ePit-Col-p21* (Exp) embryos. p21^−^ cells from Control and Exp mice were compared by 2-way ANOVA with Side and Genotype as variables (*p-*values below graphs). *n* as in panel B. (D) Length of P0 *Pit-p21* (*n =* 6–10 depending on the bone) and *Pit-tTA-p21* (*n =* 3–7) right bones, normalized to the average value of control littermates. Comparisons were done by 2-way ANOVA with Genotype and Bone identity as variables; *p-*values are shown. (E) Weight of pooled E17.5 and E18.5 *Pit-p21* (*n =* 9) and *Pit-tTA-p21* (*n =* 11) mice, normalized to the average value of control littermates and compared by unpaired 2-tailed Mann-Whitney test. (F) Left/right length ratio for femur and tibia from newborn *Pit-p21* (*n =* 10) and *Pit-tTA-p21* (*n =* 3–8) mice. Comparisons by 2-way ANOVA with Genotype and Bone identity as variables; *p-*values are shown. (G) Cell density in left and right PZ of E17.5 *Pit-p21* (*n =* 4) and *Pit-tTA-p21* (*n =* 4) mice. Comparisons were done by 2-way ANOVA with Genotype and Side as variables; *p-*values are shown below the graph (*p-*values for Sidak’s test are shown colour-coded in the graph). For panel A–G, see also S3 Data. Dox, doxycycline; E, embryonic day; EdU, 5-ethynyl-2’-deoxyuridine; Exp, experimental; PZ, proliferative zone; tTA, tetracycline transactivator.
(TIF)

S5 Fig Micro-CT measurements confirm that transient and local p21 misexpression triggers a systemic growth reduction, which is progressive and not due to leakiness in other organs. (A–B) Representative 3D reconstruction (panel A) and normalized measurements (panel B) of E17.5 bones scanned by μCT (*n =* 7 *ePit-p21*, *n* = 13 *ePit-Col-p21*). (C) Correlation analysis between the μCT measurements and the measurements done on micrographs of the same bones (*n =* 80). Spearman’s correlation coefficient (and 95% CI) is shown. (D) Left panel: weight of E15.5 and E16.5 *ePit-p21* (*n =* 10 and *n* = 5) and *ePit-Col-p21* (*n =* 11 and *n* = 6) embryos, normalized to the average control littermate and compared by 2-tailed unpaired Mann-Whitney test. Right panel: comparison of right bone length at P0. *n =* 4 *ePit-p21* and *n* = 4 *ePit-Col-p21* pups. Comparison by 2-way ANOVA with Bone and Genotype as variables. *p-*Values for Sidak’s post hoc test are shown on the graph. (E) Analysis of tdT expression in E17.5 *Pitx2-Cre/+; Col2a1-rtTA/+; Igs7*^*TRE-LSL-tdT/+*^ embryos (*Pit-Col-tdT* model, Dox at E12.5) does not reveal spurious activation outside the left cartilage templates (*n =* 2). The embryos were bisected sagittally to facilitate sectioning. For panel B–D, see S3 Data. Dox, doxycycline; E, embryonic day; LFL, left forelimb; RFL, right forelimb; tdT, tdTomato.
(TIF)

S6 Fig Transcriptomic comparison of left and right *ePit-Col-p21* cartilage. (A) Schematic of the experimental approach. After dissection and perichondrium removal, left and right cartilage elements were deprived of condyles and hypertrophic zone and were flash frozen. Left and right samples from each embryo were kept separated, and RNA was extracted for deep sequencing. (B) Unsupervised hierarchical clustering of 6 samples (left and right cartilage from 3 embryos). Note that each sample is closest to its contralateral one. (C–D) MA plot (panel C) and clustered heatmap (panel D) of the 285 DEGs (red dots in panel C) obtained by a paired DESeq2 design with adjusted *p* ≤ 0.05. (E) Normalized counts for *Cdkn1a* (*p21*) and *Rln1* (*Relaxin1*, the closest vertebrate homologue to *dilp8*) are shown for each sample. Note that *Rln1* is virtually absent from control and experimental cartilage. See also S1 Data and S2 Data. (F) Overrepresented pathways obtained from the 285 DEGs (FDR < 0.05). Note the presence of immune response pathways. (G) Normalized counts for the transcripts following a similar left–right pattern as *Cdkn1a*. The 4 examples shown are involved in cellular stress response [49–52]. For panel C, E, and G, see S1 Data and S2 Data. DEG, differentially expressed gene; FDR, false discovery rate; MA plot, log ratio (M) versus mean average (A) plot.
(TIF)

S7 Fig Potential mechanisms leading to adaptive growth after unilateral mosaic growth inhibition in long-bone chondrocytes. (A–B) Two alternative mechanisms that could underlie compensatory proliferation in response to a stress signal, classified based on whether they work at the whole growth plate level (panel A, community effect mediated by a self-propagated travelling wave) or just by proximity to the stress signal (panel B). Coloured outlines identify chondrocytes producing the stress molecule. Note that in panel A, the self-propagating signal could be the same as the original stress molecule. t1–t3 refer to subsequent times of the travelling wave. (C) Potential relay of the stress signal into circulation, which in turn impacts on placental function, causing a systemic reduction in growth (stunting).
(TIF)

## Abbreviations

BSA: bovine serum albumin
Cre: causes recombination (recombinase enzyme derived from the P1 bacteriophage)
CT: computerized tomography
DEG: differentially expressed gene
Dox: doxycycline
DRAGON: Dox-controlled and Recombinase Activated Gene OverexpressioN
E: embryonic day
EdU: 5-ethynyl-2’-deoxyuridine
IGF: insulin-like growth factor
IHH: indian hedgehog
LtSL: floxed tdTomato-STOP sequence
NK: natural killer
P: postnatal day
PFA: paraformaldehyde
PZ: proliferative zone
qRT-PCR: quantitative real-time polymerase chain reaction
RNA-seq: RNA sequencing
(r)tTA: (reverse) tetracycline transactivator
tdT: tdTomato
WT: wild-type
