# Supplementary figures and images for: Cell-nonautonomous local and systemic responses to cell arrest enable long-bone catch-up growth in developing mice
Source: PLoS Biol. 2018 Jun 26;16(6):e2005086. doi: 10.1371/journal.pbio.2005086 (PMC6019387; doi:10.1371/journal.pbio.2005086)

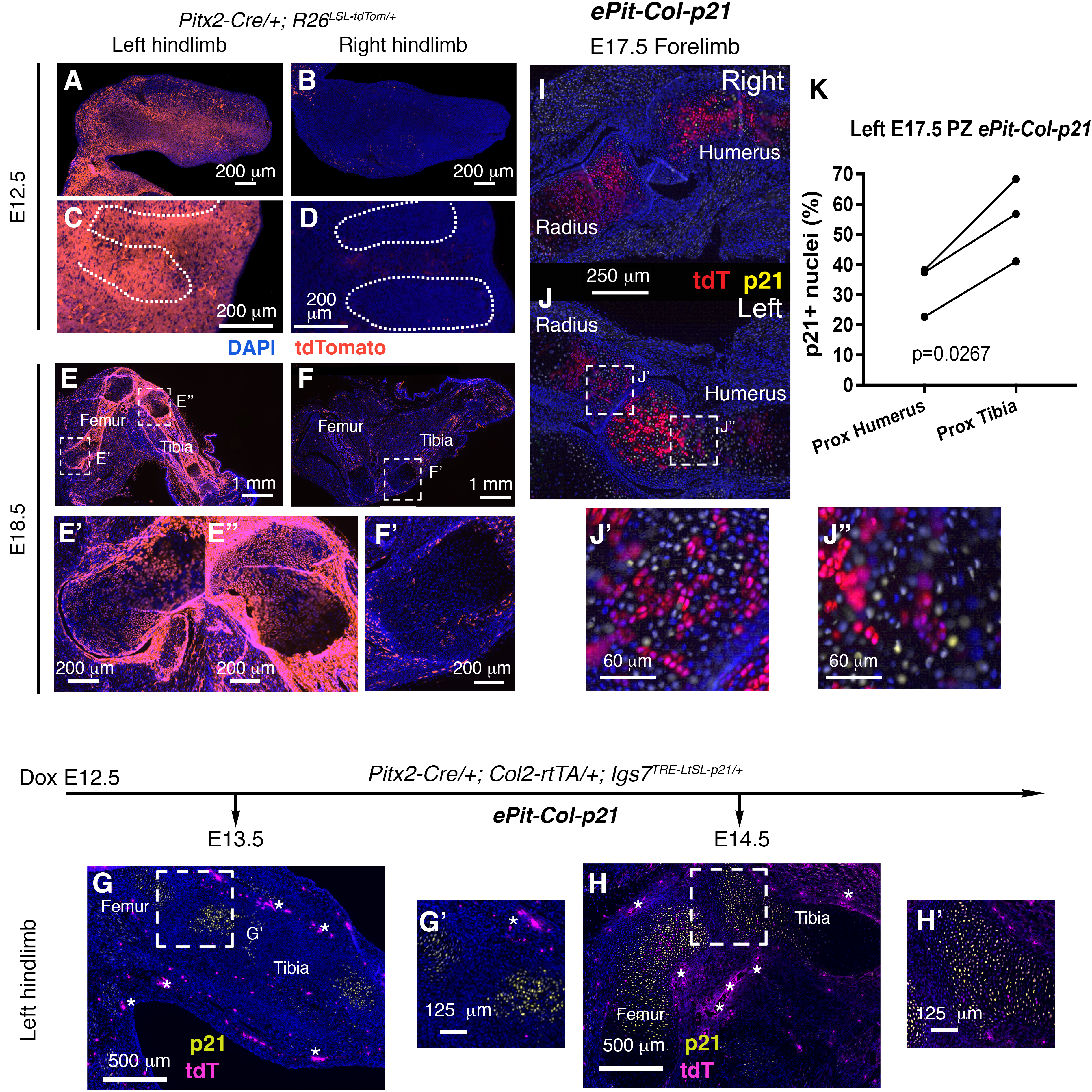

Supplement: S1 Fig — (A–F’) Pitx2-Cre females were crossed with Ai9 males to characterize the specificity of Cre-mediated labelling. Seven-μm sections from left and right hindlimbs are shown at 2 different stages: E12.5 (A–D) and E18.5 (E–F’), n = 4 for each stage. Boxed regions in panel E and panel F are shown in E’, (E”, and F’. Most of the red signal on right limbs corresponds to autofluorescent blood cells. (G–H’) Dynamics of tdT and CDKN1A (p21) activation in ePit-Col-p21 embryos, 1 d (G, G’, n = 2) and 2 d (H, H’, n = 3) after Dox administration to the pregnant female. Boxed regions in panel G and H are shown in G’ and H’. Note that activation of the transgene starts to be detectable 1 d post Dox administration, but it is not complete until 2 d post Dox. Asterisks indicate autofluorescent cells. Of note, the Pitx2-Cre allele is consistently left-predominant only when inherited from the female. (I–J”) Same as above, but E17.5 elbow sections are shown. (K) Intra-individual comparison of the proportion of p21+ nuclei in the left proximal humerus versus left proximal tibia PZ (n = 3). See also S3 Data. p-Value for 2-tailed paired t test is shown. Cre, recombinase from P1 bacteriophage; Dox, doxycycline; E, embryonic day; PZ, proliferative zone; tdT, tdTomato. (TIF) [file pbio.2005086.s005.tif]

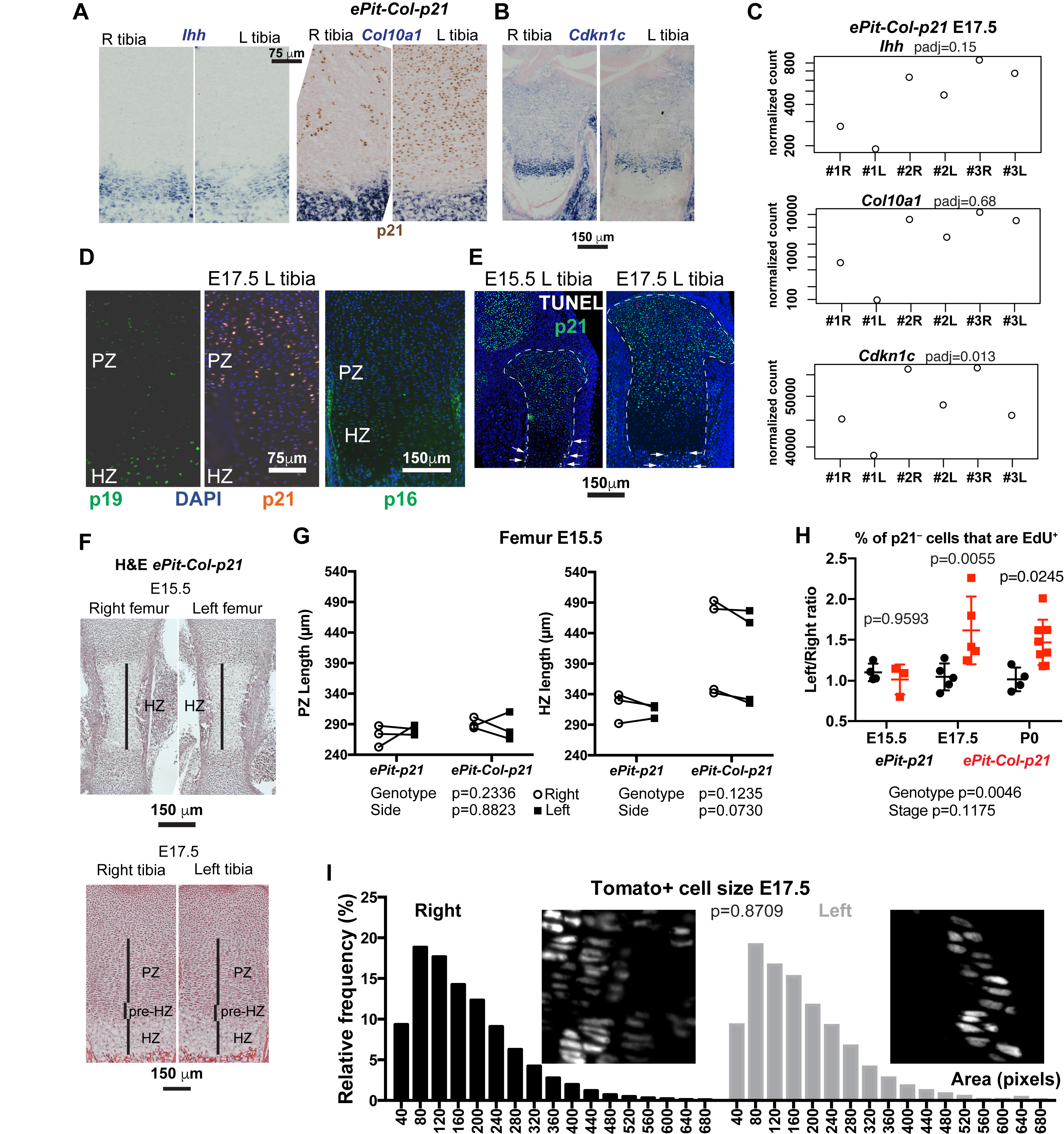

Supplement: S2 Fig — (A–C) The expression of chondrocyte maturation markers Cdkn1c, Col10a1, and Ihh is not ectopically triggered by p21 misexpression (panel A, B), but their expression is qualitatively and quantitatively diminished in the left cartilage (panel C, normalized counts and adjusted p-value from the RNA-seq experiment of S6 Fig are shown). For panel C, see S1 Data as well. (D–E) Misexpression of p21 does not lead to cell senescence in the experimental cartilage at E17.5 (panel D, monitored by p16 and p19 expression, n = 3), nor to ectopic cell death at E15.5 or E17.5 (panel E, arrows indicate TUNEL+ cells, n = 5). (F) Hematoxylin–eosin staining of E15.5 femora and E17.5 proximal tibiae from ePit-Col-p21 embryos. (G) Comparison of the length of the left and right proliferative and hypertrophic zones (PZ and HZ) of the femora from ePit-Col-p21 (n = 4) and ePit-p21 embryos (n = 3) at E15.5 (2-way ANOVA with Genotype and Side as variables was used, and p-values are shown). (H) Left/right ratios of EdU+ incorporation in the PZ of ePit-p21 and ePit-Col-p21 embryos at E15.5 (n = 4 and n = 3), E17.5 (n = 5 and n = 5), and P0 (n = 4 and n = 8). Comparison by 2-way ANOVA for Genotype and Stage (p-values below graphs). p-Values for Sidak’s post hoc test are shown in the graphs. (I) Cell size of tdT+ (i.e., p21−) chondrocytes was characterized for ePit-Col-p21 embryos at E17.5 (n = 10, see Materials and methods). Representative pictures of left and right PZ are shown. No significant difference between left and right distribution was found (p-value for 2-tailed unpaired Mann-Whitney test for ranks is shown). For panel G–I, see S3 Data. E, embryonic day; HZ, hypertrophic zone; PZ, proliferative zone; RNA-seq, RNA sequencing. (TIF) [file pbio.2005086.s006.tif]

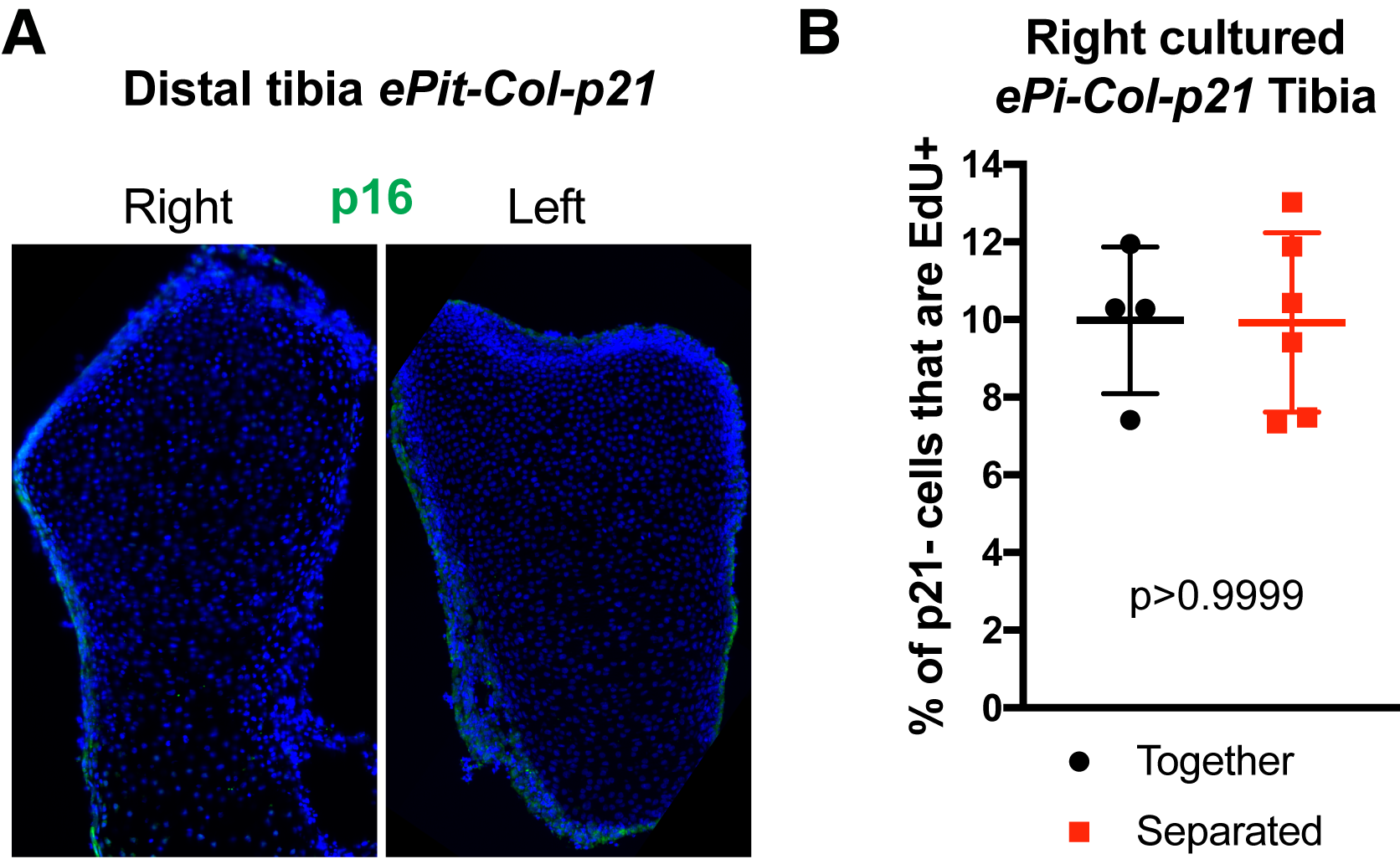

Supplement: S3 Fig — (A) After 2 d of culture, the distal tibial cartilage does not show signs of senescence, as shown by lack of p16 immunostaining (n = 3). (B) Right tibiae show the same extent of proliferation regardless of whether they are cultured together (n = 4) or separated (n = 6) from the contralateral tibia. See also S3 Data. (TIF) [file pbio.2005086.s007.tif]

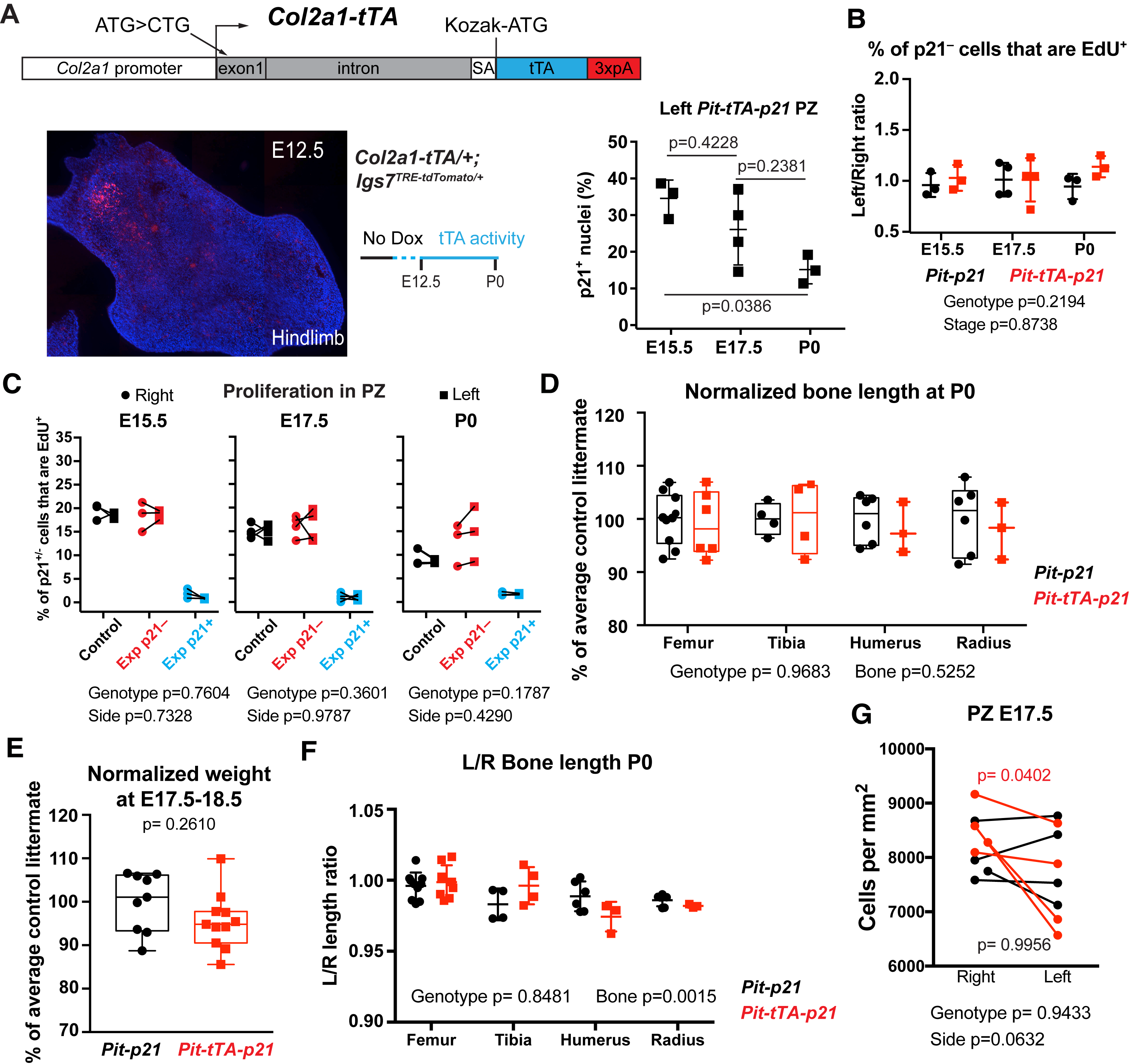

Supplement: S4 Fig — (A) Left: schematic of the new Col2a1-tTA allele. See ref. [41] for details on the Col2a1 regulatory region used. In the absence of Dox, the tTA is activated around E12.5 (detected by a germline-recombined reporter Ai62 allele) [23]. Right: percentage of p21+ chondrocytes in the PZ of left proximal tibia of Pit-tTA-p21 embryos unexposed to Dox, at E15.5, E17.5, and P0 (n = 3, 4, and 3). Comparison by 1-way ANOVA (p = 0.0368), followed by Tukey’s post hoc tests (shown). (B) Left/Right ratio of EdU incorporation in PZ chondrocytes of Pit-tTA and Pit-tTA-p21 mice at E15.5 (n = 3 each), E17.5 (n = 4 each), and P0 (n = 3 each). Comparison by 2-way ANOVA for Genotype and Stage (p-values below graphs). (C) Percentage of p21+ or p21− chondrocytes that have EdU+ nuclei in the PZ in the left and right tibias of E17.5 ePit-p21 (Control) and ePit-Col-p21 (Exp) embryos. p21− cells from Control and Exp mice were compared by 2-way ANOVA with Side and Genotype as variables (p-values below graphs). n as in panel B. (D) Length of P0 Pit-p21 (n = 6–10 depending on the bone) and Pit-tTA-p21 (n = 3–7) right bones, normalized to the average value of control littermates. Comparisons were done by 2-way ANOVA with Genotype and Bone identity as variables; p-values are shown. (E) Weight of pooled E17.5 and E18.5 Pit-p21 (n = 9) and Pit-tTA-p21 (n = 11) mice, normalized to the average value of control littermates and compared by unpaired 2-tailed Mann-Whitney test. (F) Left/right length ratio for femur and tibia from newborn Pit-p21 (n = 10) and Pit-tTA-p21 (n = 3–8) mice. Comparisons by 2-way ANOVA with Genotype and Bone identity as variables; p-values are shown. (G) Cell density in left and right PZ of E17.5 Pit-p21 (n = 4) and Pit-tTA-p21 (n = 4) mice. Comparisons were done by 2-way ANOVA with Genotype and Side as variables; p-values are shown below the graph (p-values for Sidak’s test are shown colour-coded in the graph). For panel A–G, see also S3 Data. Dox, doxycycline; E, embryonic day; [file pbio.2005086.s008.tif]

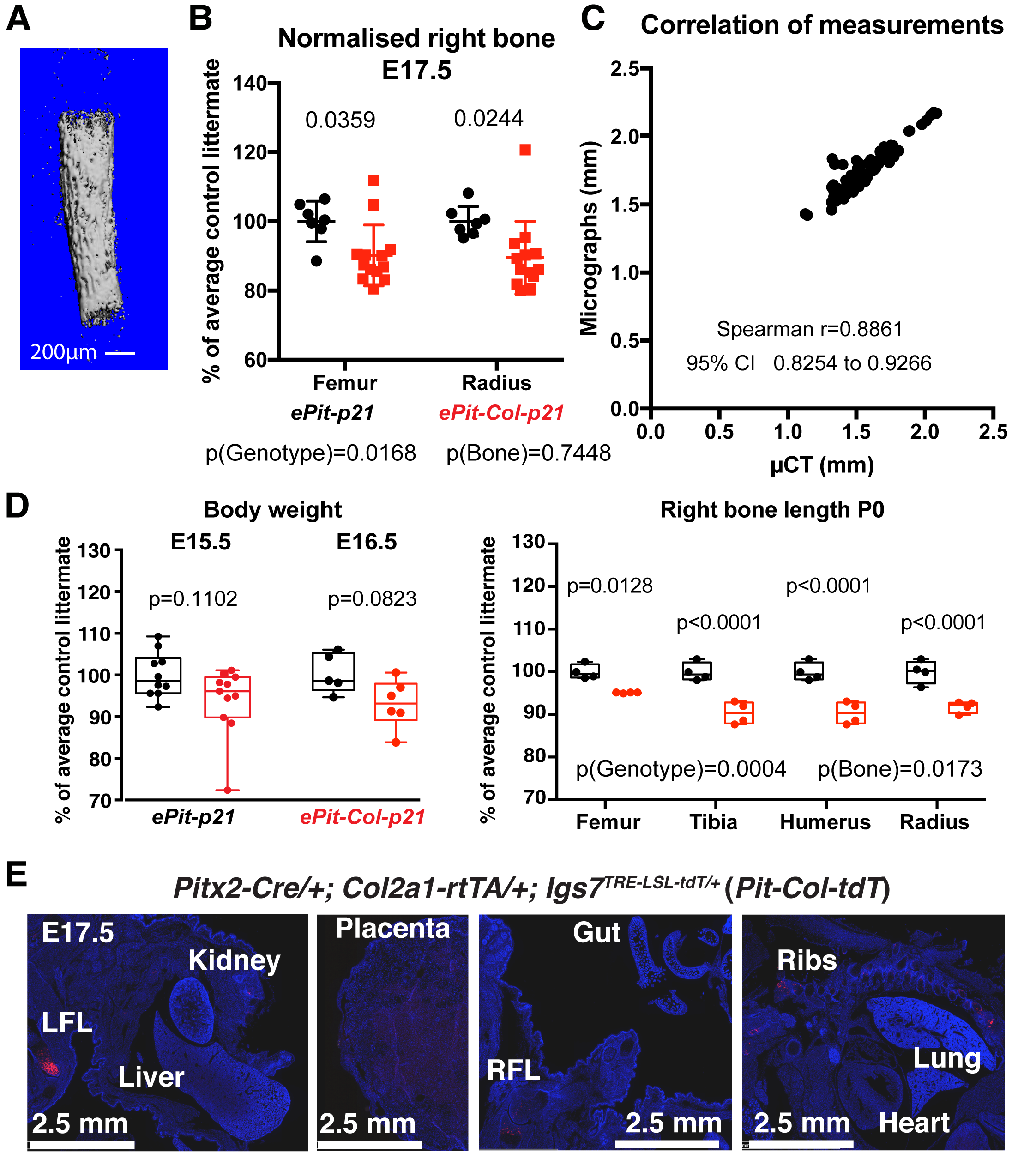

Supplement: S5 Fig — (A–B) Representative 3D reconstruction (panel A) and normalized measurements (panel B) of E17.5 bones scanned by μCT (n = 7 ePit-p21, n = 13 ePit-Col-p21). (C) Correlation analysis between the μCT measurements and the measurements done on micrographs of the same bones (n = 80). Spearman’s correlation coefficient (and 95% CI) is shown. (D) Left panel: weight of E15.5 and E16.5 ePit-p21 (n = 10 and n = 5) and ePit-Col-p21 (n = 11 and n = 6) embryos, normalized to the average control littermate and compared by 2-tailed unpaired Mann-Whitney test. Right panel: comparison of right bone length at P0. n = 4 ePit-p21 and n = 4 ePit-Col-p21 pups. Comparison by 2-way ANOVA with Bone and Genotype as variables. p-Values for Sidak’s post hoc test are shown on the graph. (E) Analysis of tdT expression in E17.5 Pitx2-Cre/+; Col2a1-rtTA/+; Igs7TRE-LSL-tdT/+ embryos (Pit-Col-tdT model, Dox at E12.5) does not reveal spurious activation outside the left cartilage templates (n = 2). The embryos were bisected sagittally to facilitate sectioning. For panel B–D, see S3 Data. Dox, doxycycline; E, embryonic day; LFL, left forelimb; RFL, right forelimb; tdT, tdTomato. (TIF) [file pbio.2005086.s009.tif]

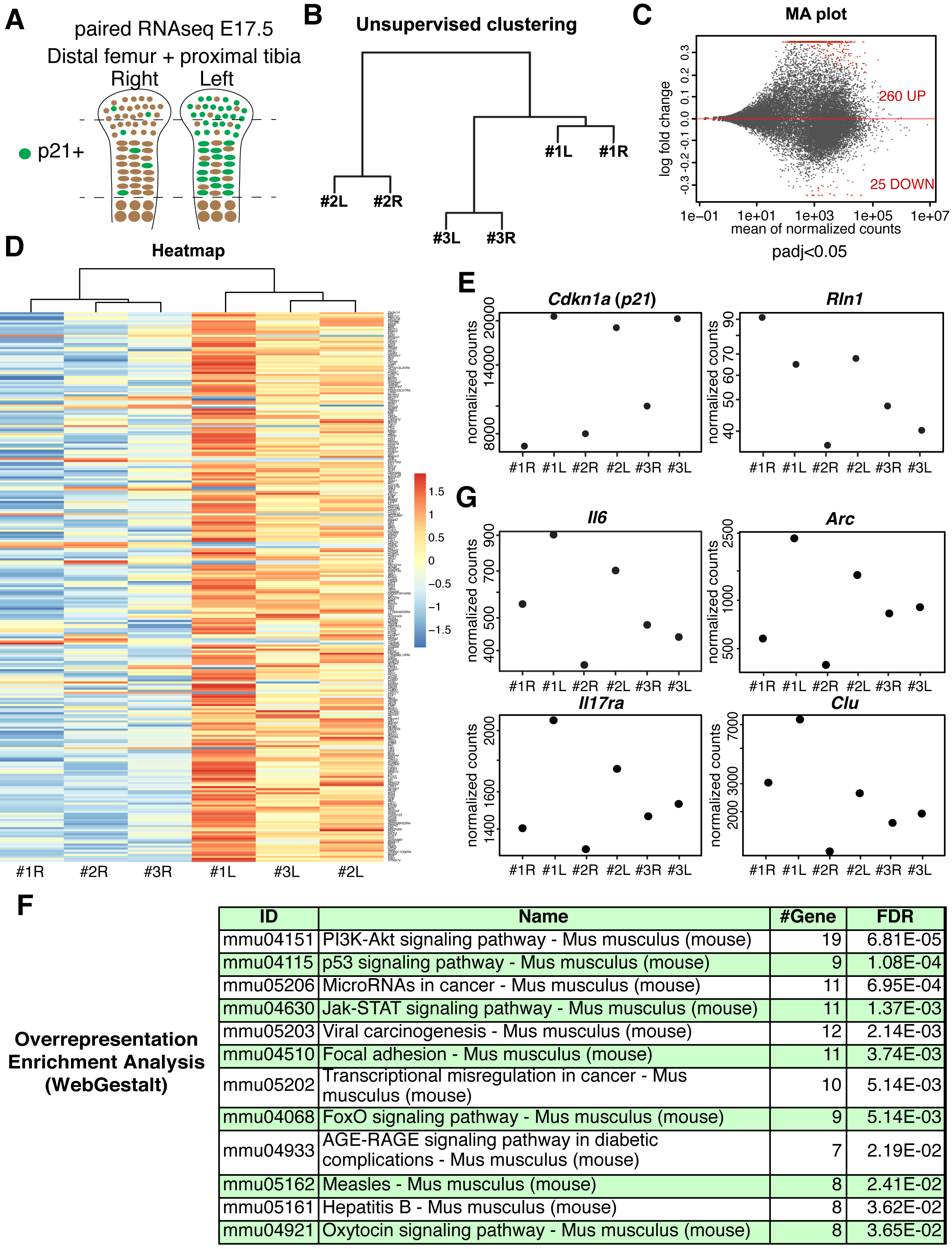

Supplement: S6 Fig — (A) Schematic of the experimental approach. After dissection and perichondrium removal, left and right cartilage elements were deprived of condyles and hypertrophic zone and were flash frozen. Left and right samples from each embryo were kept separated, and RNA was extracted for deep sequencing. (B) Unsupervised hierarchical clustering of 6 samples (left and right cartilage from 3 embryos). Note that each sample is closest to its contralateral one. (C–D) MA plot (panel C) and clustered heatmap (panel D) of the 285 DEGs (red dots in panel C) obtained by a paired DESeq2 design with adjusted p ≤ 0.05. (E) Normalized counts for Cdkn1a (p21) and Rln1 (Relaxin1, the closest vertebrate homologue to dilp8) are shown for each sample. Note that Rln1 is virtually absent from control and experimental cartilage. See also S1 Data and S2 Data. (F) Overrepresented pathways obtained from the 285 DEGs (FDR < 0.05). Note the presence of immune response pathways. (G) Normalized counts for the transcripts following a similar left–right pattern as Cdkn1a. The 4 examples shown are involved in cellular stress response [49–52]. For panel C, E, and G, see S1 Data and S2 Data. DEG, differentially expressed gene; FDR, false discovery rate; MA plot, log ratio (M) versus mean average (A) plot. (TIF) [file pbio.2005086.s010.tif]

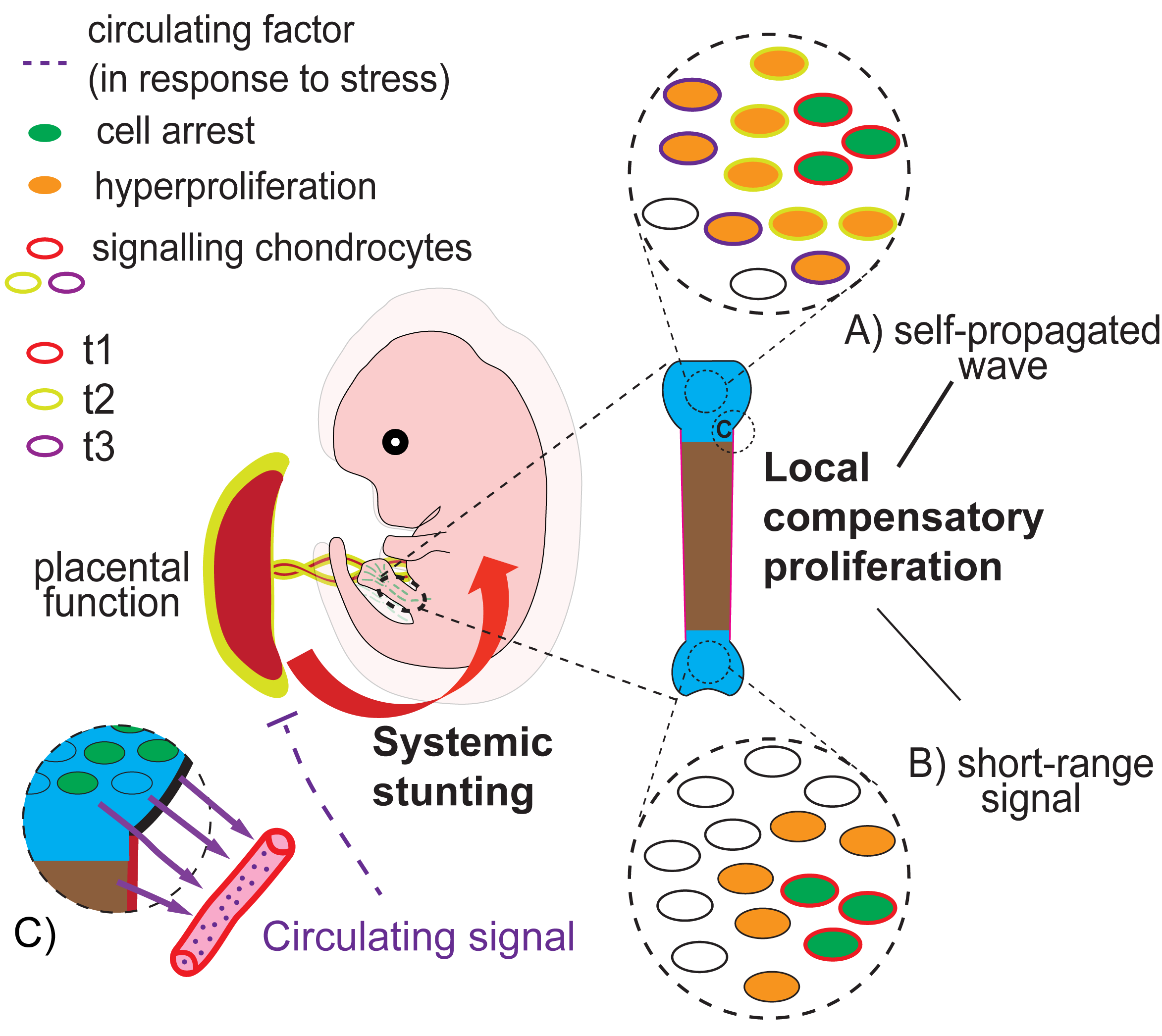

Supplement: S7 Fig — (A–B) Two alternative mechanisms that could underlie compensatory proliferation in response to a stress signal, classified based on whether they work at the whole growth plate level (panel A, community effect mediated by a self-propagated travelling wave) or just by proximity to the stress signal (panel B). Coloured outlines identify chondrocytes producing the stress molecule. Note that in panel A, the self-propagating signal could be the same as the original stress molecule. t1–t3 refer to subsequent times of the travelling wave. (C) Potential relay of the stress signal into circulation, which in turn impacts on placental function, causing a systemic reduction in growth (stunting). (TIF) [file pbio.2005086.s011.tif]
